# Substrate geometry affects population dynamics in a bacterial biofilm

**DOI:** 10.1073/pnas.2315361121

**Published:** 2024-04-15

**Authors:** Witold Postek, Klaudia Staśkiewicz, Elin Lilja, Bartłomiej Wacław

**Affiliations:** ^a^Dioscuri Centre for Physics and Chemistry of Bacteria, Institute of Physical Chemistry, Polish Academy of Sciences, Warszawa 01-224, Poland; ^b^Broad Institute of Massachusetts Institute of Technology and Harvard, Cambridge, MA 02142; ^c^School of Physics and Astronomy, The University of Edinburgh, Edinburgh EH9 3FD, United Kingdom

**Keywords:** biofilms, evolution of bacteria, microfluidics

## Abstract

Biofilms inhabit a range of environments, such as dental plaques or soil micropores, often characterized by noneven surfaces. However, the impact of surface irregularities on the population dynamics of biofilms remains elusive, as most experiments are conducted on flat surfaces. Here, we show that the shape of the surface on which a biofilm grows influences genetic drift and selection within the biofilm. We culture *E*scherichia* coli* biofilms in microwells with a corrugated bottom surface and observe the emergence of clonal sectors whose size corresponds to that of the corrugations, despite no physical barrier separating different areas of the biofilm. The sectors are remarkably stable and do not invade each other; we attribute this stability to the characteristics of the velocity field within the biofilm, which hinders mixing and clonal expansion. A microscopically detailed computer model fully reproduces these findings and highlights the role of mechanical interactions such as adhesion and friction in microbial evolution. The model also predicts clonal expansion to be limited even for clones with a significant growth advantage—a finding which we confirm experimentally using a mixture of antibiotic-sensitive and antibiotic-resistant mutants in the presence of sublethal concentrations of the antibiotic rifampicin. The strong suppression of selection contrasts sharply with the behavior seen in range expansion experiments in bacterial colonies grown on agar. Our results show that biofilm population dynamics can be affected by patterning the surface and demonstrate how a better understanding of the physics of bacterial growth can be used to control microbial evolution.

## Significance

Biological evolution in a biofilm—a conglomerate of microorganisms adhering to a surface—can be undesirable, resulting in antibiotic resistance or the disruption of genetically engineered bacterial communities utilized in industry. Here, we demonstrate how to impede these processes by leveraging the physics of the biofilm. We introduce fluorescent bacteria into patterned microwells with a corrugated surface and track their progeny using microscopy. We show that fluorescent sectors emerge in close correspondence to the surface pattern, and the surface corrugations inhibit the spread of antibiotic-resistant bacteria in the biofilm during antibiotic treatment. These findings suggest a passive method to curb the spread of unwanted mutations within biofilms, carrying valuable implications for both medicine and industry.

Bacterial biofilms are conglomerates of cells bound together by extracellular matrix containing compounds such as polysaccharides and nucleic acids (1). Found widely in natural ecosystems, biofilms play crucial roles in medicine (2, 3) and technology (4, 5). In all these contexts, the emergence of new genetic variants is a concern. For example, cells in a biofilm can acquire resistance to antibiotics through various mechanisms (6, 7), including de novo genetic mutations and horizontal gene transfer (8). Limiting the spread of such new variants is desirable, aligning with ongoing efforts to control biological evolution, which are gaining momentum (9).

The growth and population dynamics of biofilms are influenced by biochemical cues (10), competition (11), cooperation (12), cell death (13), and mechanical interactions (14, 15). In particular, the role of cell–cell and cell–surface interactions in the establishment of new variants has been explored in experimental (16, 17) and theoretical (18, 19) studies of colonies growing on agarose gel surfaces. In such colonies, bacteria primarily replicate at the expanding front due to nutrient depletion and waste accumulation at the center (20). When a new mutant arises at the front, it either “surfs” along the advancing front and forms a clonal cluster that expands into a new territory or it gets outpaced by neighboring clonal populations and loses the competition for nutrients (16, 21–25). The probability of a new variant spreading depends on the interplay of mechanical interactions between bacterial cells and with the substrate, as well as the variant’s fitness compared to the parent strain (13, 20, 26–33). These conclusions hold true also for three-dimensional, thick biofilms, which consist of a growing active layer and a quiescent bulk (34, 35).

A significant limitation in these studies has been the use of flat substrates. In natural environments, biofilms often grow on nonflat surfaces such as rocks and underwater stones (36), pipelines (4), mineralized surface of urinary catheters (37), pores of the human skin (38), porous beads in water treatment plants (39), marine snow (40), and colon crypts (41). Recent work on bacterial colonies growing on rough agar has shown that selection decreases and neutral drift increases compared to growth on flat agar (42). A similar effect has been obtained by placing obstacles in the path of an expanding colony (43). Clonal dynamics is also affected in colonies encountering physical barriers during expansion (44). However, these studies primarily focus on quasi-two-dimensional colonies that grow parallel to the rough surface, which does not consider the perpendicular growth of 3D biofilms. Consequently, there is little experimental and in silico research on the influence of surface irregularities on the biofilm population dynamics (45).

To bridge this gap, we employ a microfluidics-based model system to investigate a more realistic scenario of a biofilm growing on a rough surface, where the top of the biofilm is sheared off by flow. We aim to understand how the population dynamics of genetic variants is influenced by substrate roughness. Specifically, we grow biofilms initiated with a mixture of fluorescently labeled cells in microscopic wells, featuring sine-like undulations of the bottom surface. We show that a moderate level of surface corrugation at the scale slightly larger than the cell size reduces both genetic drift and selection strength, enabling coexistence of clones with significantly different growth rates. This holds true even when the amplitude of substrate roughness is much smaller compared to the biofilm thickness. We attribute this effect to cell–surface interactions that influence cell orientation and movement in the biofilms. The resulting velocity field hinders clonal mixing everywhere in the biofilm except close to the substrate, where bacteria can orientate and move parallel to the surface. Substrate roughness strongly limits this movement, resulting in narrow clonal sectors that do not invade each other even when clones have different fitness. Our microfluidic experiments, coupled with mathematical modeling, underscore the role of mechanical interactions in biofilm growth and evolution and suggest a simple and robust way of controlling biofilm genetic diversity by varying substrate roughness.

## Results

### Substrate Roughness Leads to Clonal Sectors Mirroring the Substrate Geometry.

**Fig. 1.**
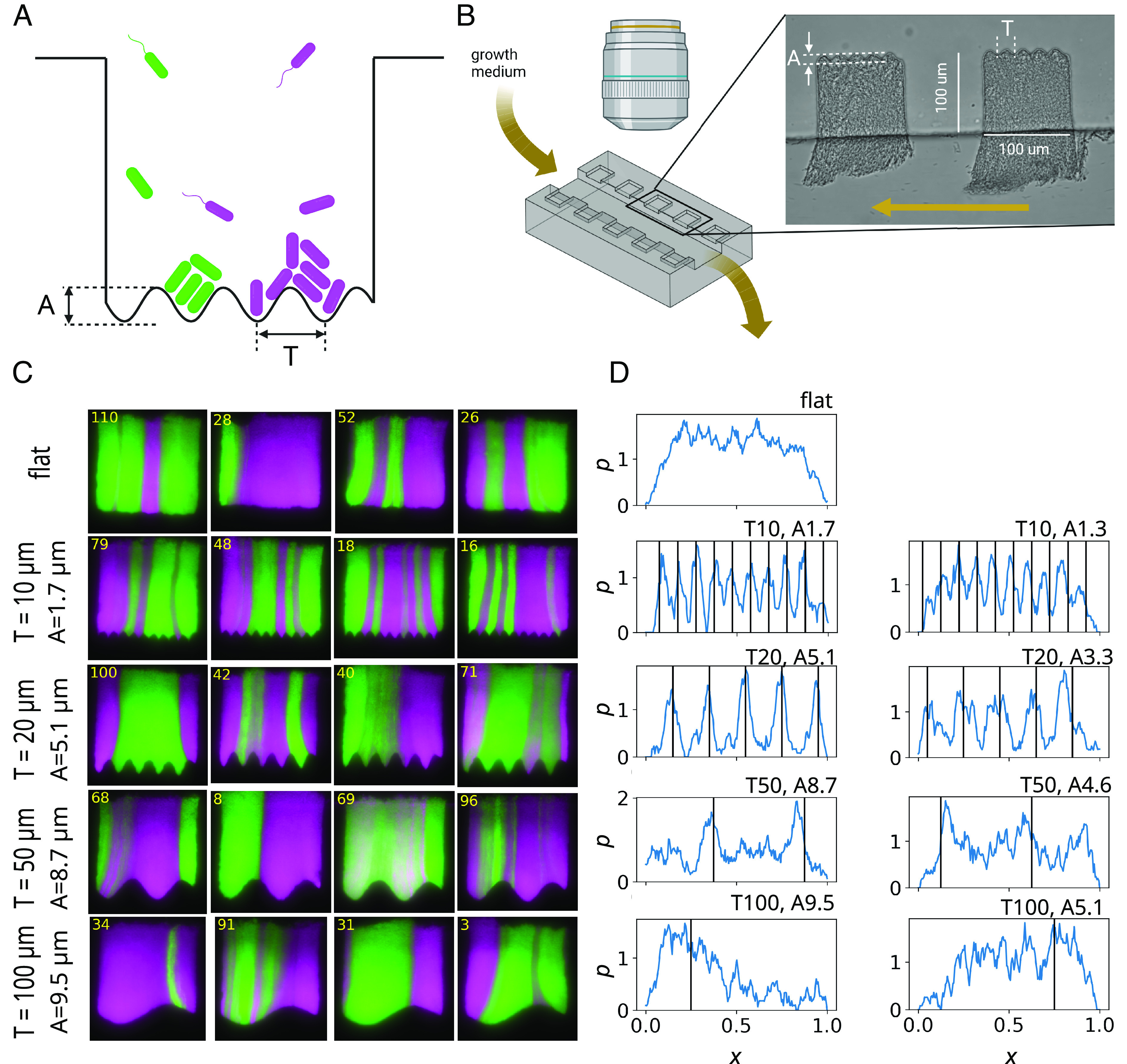
Substrate geometry affects the number and size of clonal sectors. (*A*) Illustration of a single 100 × 100 µm well with a corrugated bottom for biofilm growth. (*B*) A simplified diagram of the experimental setup. The actual microfluidic device has 240 wells with sine-like corrugations of eight different amplitudes A and periods T, each replicated 20 times, along with 80 flat-bottomed wells. (*C*) End-point snapshots (t=143 h) of randomly selected wells with different configurations. Clonal sectors form preferentially in the grooves of the bottom surface. Fluorescent strains mKate and GFP have been rendered in magenta and green, respectively. (*D*) End-point probability density of finding a sector boundary at normalized position x∈[0,1] inside the well, averaged over three biological replicates, for different well types. Black vertical lines show positions of the ridges of the corrugations. All (T,A) are in μm.

We cultured biofilms in a microfluidic device (Fig. 1 *A* and *B* and *SI Appendix*, Fig. S1) comprising multiple microwells connected to a deeper main channel for delivering growth medium and bacteria to the wells, similar to previously published devices (46, 47). However, in contrast to those studies, each well (100 × 100 × 7 µm) had a sine-patterned bottom surface (Fig. 1*A*), with varying amplitude and period across different wells, including some with flat bottoms. The amplitude of surface height modulation was only a small fraction of the total well height, and the sine-like indentations did not physically separate different regions of the biofilm. The quasi-two-dimensional well shape allowed for the formation of multilayered biofilms similar to naturally occurring biofilms, but the biofilms remained thin enough for cell movement to be limited mostly to the XY plane. This facilitated imaging of the biofilm using a wide-field epifluorescent microscope.

To examine the impact of growth on corrugated surfaces on clonal dynamics within the biofilm, we performed standing variation experiments using the biofilm-forming strain *E*scherichia* coli* 83972 and its derivatives (*Materials and Methods*). We inoculated the microfluidic device with a 1:1 mixture of similarly fit (**SI Appendix*, Methods*) red (mKate) and green (GFP) fluorescent cells. Nutrient broth (LB) was pumped through the main channel, while we monitored all wells using fluorescence and phase-contrast microscopy. The experiment was conducted at room temperature (25 °C) to reduce growth and clogging in the main channel. Nutrient broth flow trimmed excess biofilm extending into the main channel, thus allowing nutrients to diffuse into the wells. Biofilms quickly formed in all wells, and the initial uniform distribution of bacteria (**SI Appendix*, Methods*) developed into fluorescent sectors aligned with the direction of biofilm growth. The number of sectors and their positions matched the undulating pattern of the well bottom (Fig. 1*C* and Movie S1), especially evident for undulations with periods much smaller than the well’s width. Most sectors were parallel to the side walls, but some were slightly bent, suggesting nonuniformities in the velocity field within the well.

To quantify the difference between sector patterns in different well types, we calculated the probability distribution of finding the boundary between adjacent sectors at position x in the well, at the end point of the experiment. Fig. 1*D* shows that many of these distributions are nonuniform, with peaks positioned near the ridges of the corrugations. These distributions stabilize within the first 2 d of biofilm growth and change little afterward (*SI Appendix*, Fig. S2). We also calculated the mean sector size as a fraction of the well’s width (*Materials and Methods* and **SI Appendix*, Methods*): *SI Appendix*, Fig. S3 *A* and *B* shows that long-period corrugations result on average in larger sectors compared to flat-bottomed and short-period ones, but only if the amplitude of corrugations is sufficiently large. Once established, the sectors maintained nearly constant width during live imaging (*SI Appendix*, Fig. S3*G*).

In contrast to similar experiments in bacterial colonies (16, 18, 21, 24, 25), the fluorescence within the sectors appeared more heterogeneous, indicating that the sectors were not entirely monoclonal. We quantified this diversity using mean heterozygosity $H=f_{1}f_{2}/\left(f_{1}+f_{2}\right)$ calculated along the line near the bottom of the well, where $f_{1}\mathrm{and}f_{2}$ are the frequencies of the two strains obtained from their fluorescence intensities (*SI Appendix*, Fig. S4*A*). H=0.25 if both strains are mixed in equal proportions, whereas H=0 if only one strain is present. *SI Appendix*, Fig. S4*B* shows the heterozygosity as a function of time, averaged over all wells of the same type. While the strains appear to be initially mixed (H≈0.22), heterozygosity decreases in time. This is particularly pronounced for wells with $\left(T,\;A\right)=\left(50,\;8.7\right),\;\left(20,\;5.1\right),\;\left(10,\;1.7\right)$ μm, for which Fig. 1 showed well-delineated clonal sectors established in the grooves of the corrugated bottoms. This indicates that while both strains may coexist in some grooves, clonal expansion reduces within-the-pocket diversity, while corrugations hinder the mixing of adjacent clones.

### Bent Sectors Arise from the Nonuniformity of the Velocity Field Caused by Heterogeneous Adhesion.

**Fig. 2.**
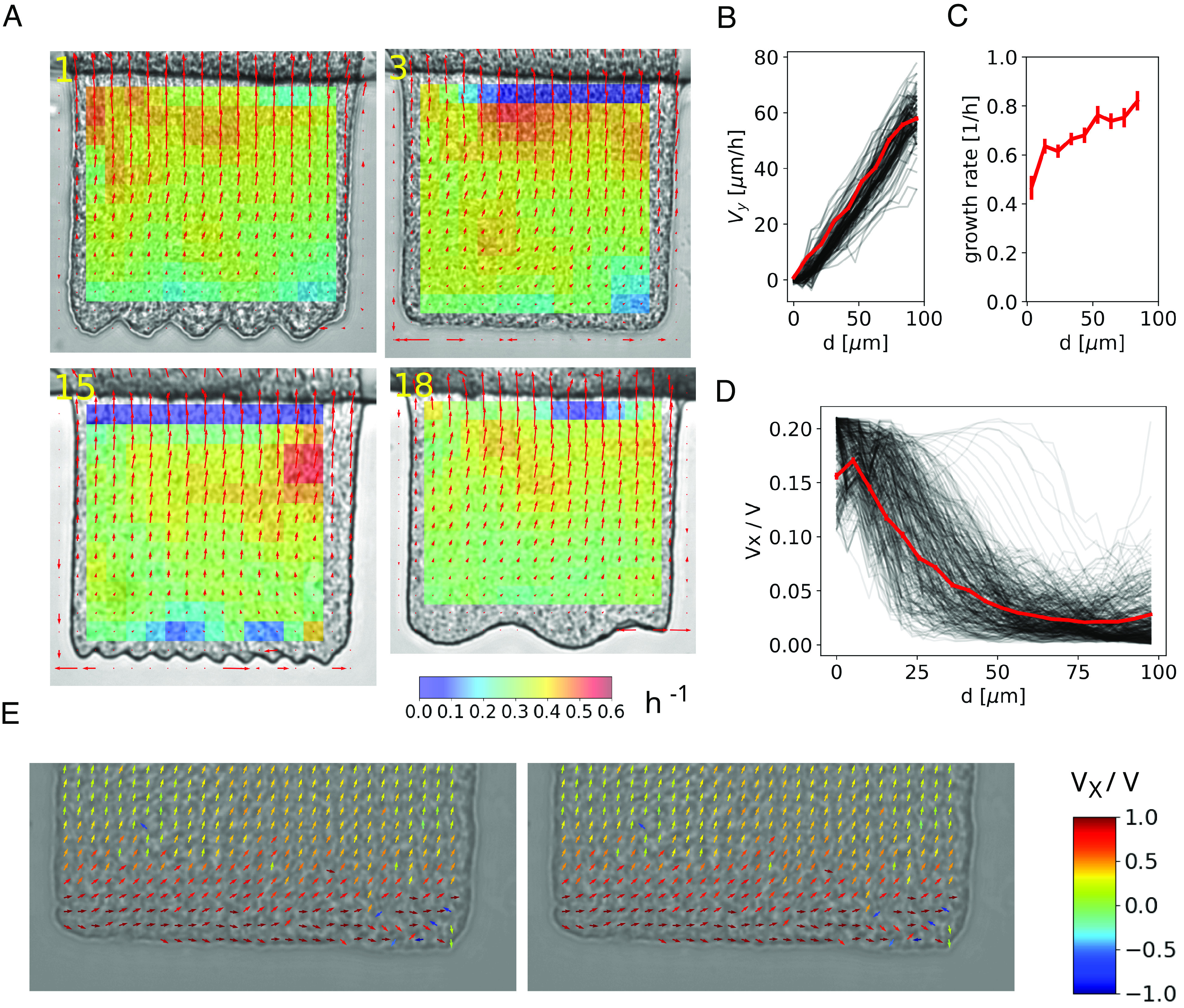
Velocity field in the wells. (*A*) Examples of the velocity field (arrows) and growth rate (color map) for wells of different types. (*B*) Average vertical velocity (red curve) as a function of distance d from the bottom surface of flat-bottomed wells (n=11), for an 18 h old biofilm. The individual black lines represent velocities measured at various horizontal positions (approx. 10 per well) within the well, for 11 wells. (*C*) Growth rate versus distance d from the bottom, for the same data in panel *C*. Error bars = SEM. (*D*) Average horizontal component of the velocity field (red curve) versus d. The black lines are as in panel *B*. (*E*) Zoomed-in plots of the velocity field near the bottom for a corrugated- and a flat-bottom well. Arrows are color-coded based on the $v_{x}/v$ component of the field (scale bar).

Some fluorescent sectors in Fig. 1*C* are bent, which suggest nonuniform flows in the biofilm (see also Movie S1). This nonuniformity could be a result of differences in the growth rate or variations in adhesion to the surface in different parts of the biofilm. To distinguish between these two possibilities, we calculated the velocity field in the biofilm from short bright-field time-lapse videos recorded at a higher frame rate (1 fps) than those used for Fig. 1, using an optical flow–based method (**SI Appendix*, Methods*). We then calculated the local growth rate g(x,y) using the continuity equation for an incompressible fluid with local mass generation: $g\left(x,y\right)=\vec{\nabla }\cdot \vec{v}\left(x,y\right)$. Fig. 2*A* shows the velocity field superimposed on the field representing the growth rate for selected wells. It is evident that except for a small gradient in the direction of growth (the vertical direction in the picture), the growth rate remains relatively uniform, even though the velocity field occasionally has a significant horizontal (perpendicular to the growth direction) component. This suggests that most of the observed heterogeneities of the velocity field are due to stronger adherence of parts of the biofilm to the sides of the wells.

### Spatial Isolation Arises from the Focusing Property of the Velocity Field.

Next, we investigated the properties of the velocity field that could explain the spatial isolation of the sectors. Fig. 2*B* shows the vertical component of the velocity field, averaged across all horizontal positions of 11 flat-bottom wells. The near-linear increase of the vertical velocity with distance from the bottom indicates that the growth rate in our system is similar at all depths inside the biofilm (Fig. 2*C*). This is true for biofilms of different ages and positions in the device, although the velocity gradient (equal to the growth rate) decreases in time as the biofilms mature (*SI Appendix*, Figs. S5 and S6). Such a velocity field facilitates the orientation of rod-shaped cells in the direction of growth (48). Fig. 2*D* shows that the horizontal component of the velocity field, plotted as a fraction of the total velocity, decreases rapidly with distance from the bottom. Consequently, significant horizontal cell movement occurs primarily near the bottom surface of the well. This movement enhances genetic drift, promoting the establishment of a single clone in flat-bottom wells. To assess whether the presence of surface corrugations hinders this movement, we examined the velocity field near the bottom of flat and corrugated wells. Fig. 2*E* shows that the horizontal movement of cells is indeed disrupted by the presence of surface corrugations.

### The Computer Model Qualitatively Reproduces the Experimental Results.

**Fig. 3.**
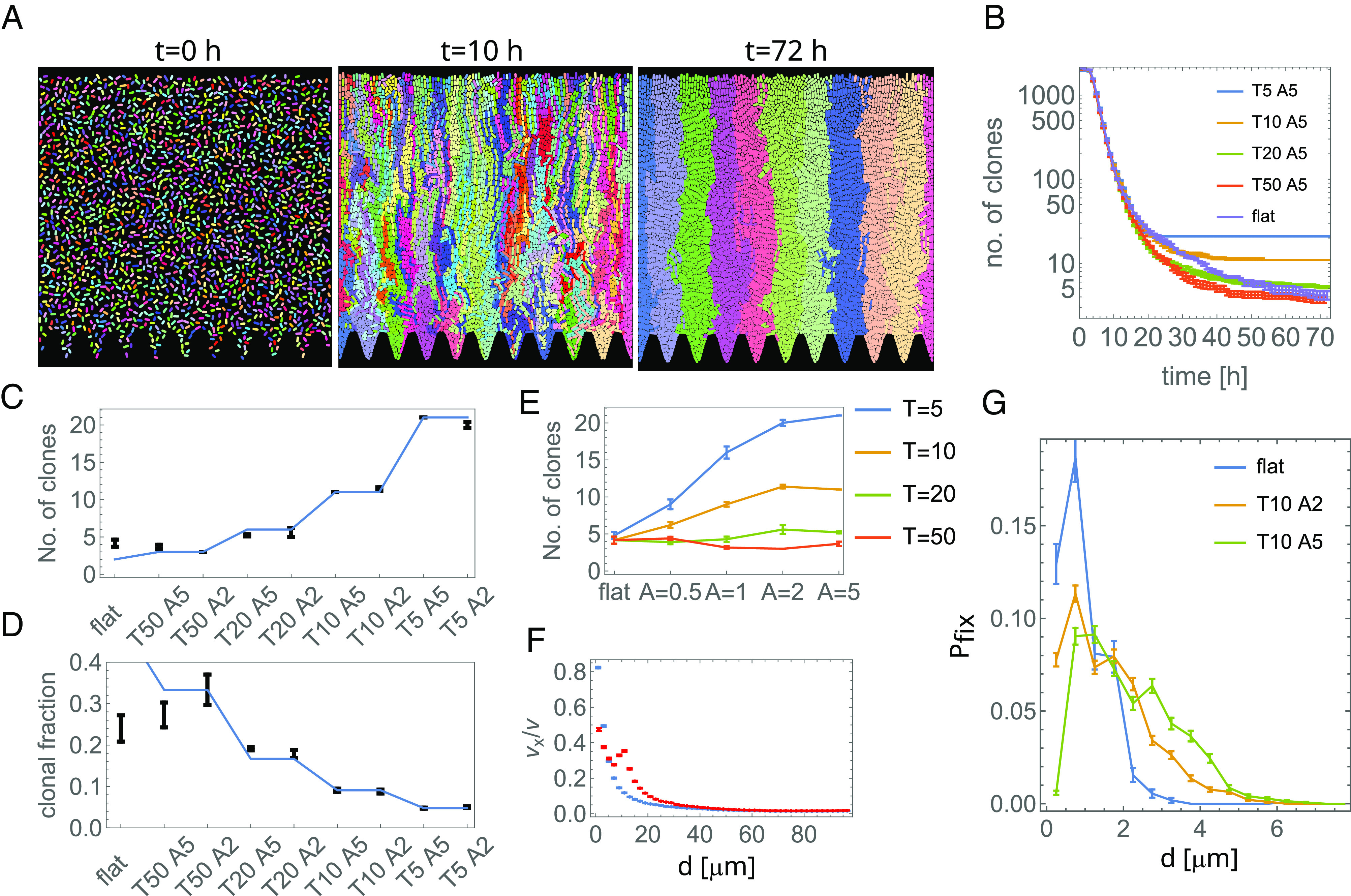
Computer model of the experiment. (*A*) Simulation snapshots for a well with T=10 μm,A=5 μm. Colors represent different clones. (*B*) The number of clones as a function of time, for different T,A. (*C* and *D*) The number of clones after t=72 h (*C*) and the mean fraction of the population occupied by a clone (*D*), for different T,A. Points are the results of computer simulations, the blue line is the theoretically predicted average number of clones $N_{\text{clones}}=(T+100\;\mu m)/T$ and the fraction occupied by a single clone $f=1/N_{clones}$ based the number of pockets, under the assumption of intrapocket clonality. (*E*) The number of clones (as in panel *C*) as a function of amplitude A, for different periods T. (*F*) The horizontal component of the local velocity field as a function of the distance from the bottom. The blue points are for a flat bottom well, and the red points are for (T,A)=(10,5) μm. (*G*) Probability density that the progeny of a cell, originated at t=2 h at a distance d from the bottom, survives until t=72 h. In all panels, error bars = SEM, and T,A values are in μm.

To understand the experimental results, we used a 2D computer model similar to the one reported before (20) to simulate a colony of rod-shaped bacteria in a square well with one open side, through which cells could escape and be removed from the system. For simplicity, we assumed that all bacteria in the biofilm divided at equal rates (cf. Fig. 2*C*) and neglected adhesion to the substrate. To facilitate the tracking of the clonal sectors, the system was initialized with bacteria each assigned a different, heritable genotype, represented by a random color. The simulation ran for 72 h for different pairs of A,T. Fig. 3*A* shows that as expected, corrugated bottom surface constrained the spread of genetic variants to sectors originating from within each sine-like pocket. The number of sectors decreased in time but eventually stabilized for corrugated surfaces (Fig. 3*B*). The final number of sectors closely aligned with the number of undulations (Fig. 3 *C* and *D*), for a broad range of amplitudes A (Fig. 3*E*). In contrast, a flat or only slightly corrugated bottom surface allowed bacteria to spread along it, leading to increased genetic drift. Fig. 3*F*, which is analogous to the experimental Fig. 2*D*, shows that horizontal movement was indeed limited near the bottom surface. A new clonal variant could only establish if initiated by a cell close to the substrate on which the biofilm grows (Fig. 3*G*); otherwise, the variant would be pushed out of the well by the replicating cells that were closer to the substrate. This is the opposite of what occurs in bacterial colonies growing on a flat agar surface, where new variants can only establish if they emerge close to the colony edge (18, 20).

### Model Prediction that Surface Corrugations Suppress Selection Is Confirmed Experimentally.

**Fig. 4.**
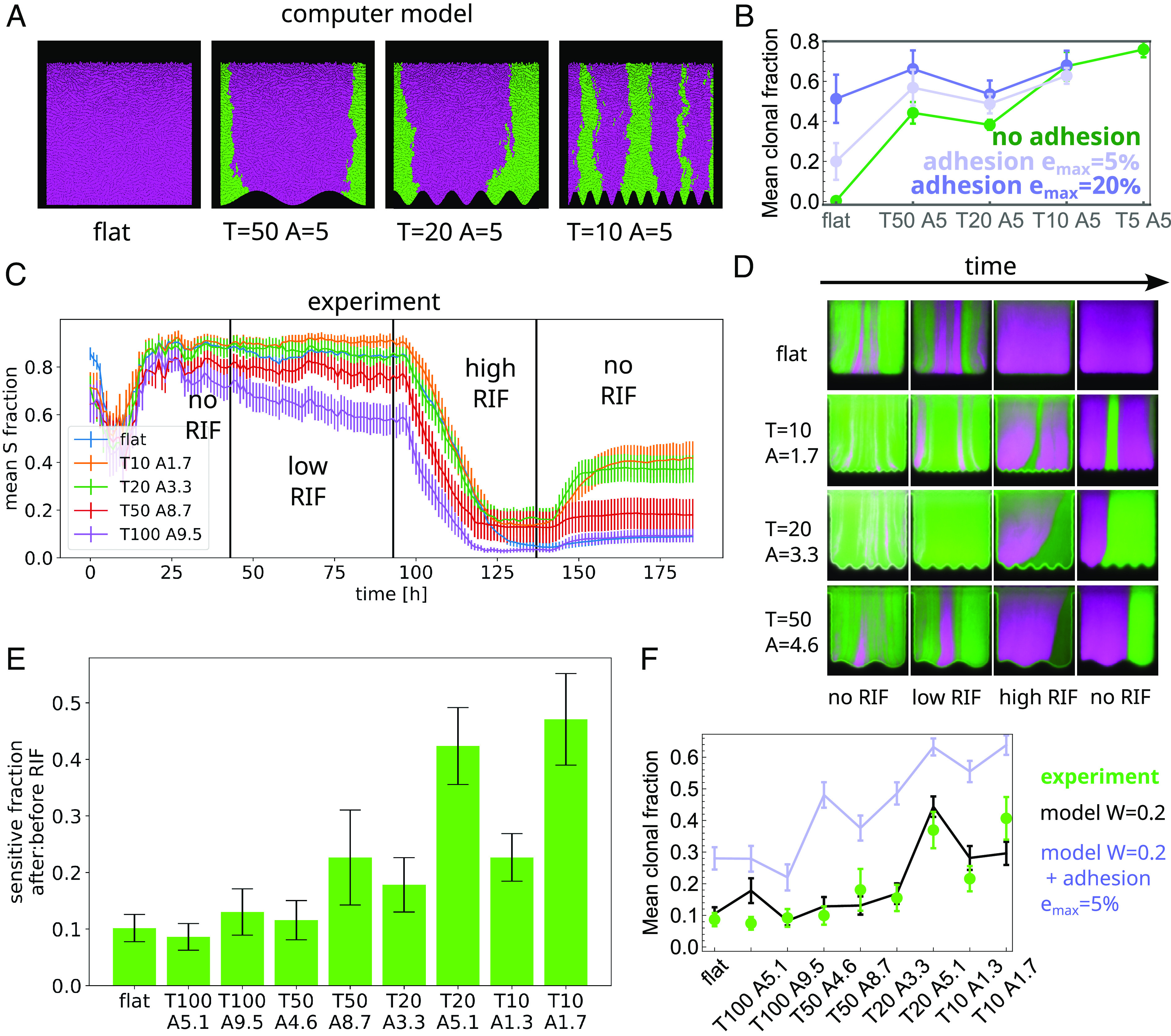
Corrugated surface limits selection. (*A*) Computer simulation snapshots (t=72 h) illustrate how corrugations constrain the spread of the fitter strain (magenta, relative fitness $W_{R/S}=1.5$ compared to the green strain). (*B*) In the model, the fraction of the less-fit strain remaining in the biofilm after 72 h increases as the corrugation period T decreases, for absent or weak adhesion ($\epsilon_{\text{max}}=5\%$). Strong adhesion ($\epsilon_{\text{max}}=20\%$) nullifies this effect. (*C*) Experimental validation of the model: the fraction of RIF-sensitive green strain as a function of time in an experiment in which the strength of selection was varied in time by adjusting the RIF concentration. The resistant strain dominated in flat-bottomed and large-T wells after transient RIF exposure, while rapid undulations (small T) significantly limited its spread. (*D*) Snapshots of wells corresponding to different phases of the experiment from panel *C*. Magenta and green are resistant and sensitive strains, respectively. (*E*) The ratio of the sensitive strain fractions at two time points: after (t=182 h) and before (t=41 h) the exposure to RIF. (*F*) The mean sensitive clonal fraction at the end of the experiment (t=182 h) in different types of wells (green points) is reproduced by the computer model without adhesion (black line). Strong adhesion worsens the agreement (pale blue line). Error bars are SEM, and T,A are in μm. Data in panels *C*–*F* come from a single biological replicate.

So far, we considered neutral variants, i.e., all genotypes (both in the experiment and in the model) had nearly identical growth rates. Since spatial patterning of the bottom surface decreases interactions between neighboring populations of bacteria, we reasoned that it should also prevent fitter variants from taking over. To test this hypothesis, we initialized our computer model with a 10:1 mixture of normal-growing to faster-growing clones with relative fitness W=1.5 (*Materials and Methods*) and measured the fraction of the less-fit clone after t=72 h. Fig. 4*A* shows that the less-fit clone is quickly outcompeted by the faster-growing variant in flat-bottomed wells, but corrugations impede its spreading, resulting in a substantial fraction (40 to 60%) of the less-fit clone remaining (Fig. 4*B*). Moreover, when simulated bacteria adhere to the surface of the well, the effect of selection is further diminished (pale blue and violet points in Fig. 4*B*). Therefore, our system acts as a suppressor of selection (49), allowing coexistence of diverse genetic variants.

To experimentally test this prediction, we inoculated our microfluidic device with a 1:10 mixture of mKate RIF^R^ red fluorescent, rifampicin (RIF)-resistant strain and GFP RIF^S^ green fluorescent rifampicin-sensitive strain. In the absence of RIF, the resistant strain had a small fitness disadvantage ($W_{R/S}\approx 0.8$) compared to the sensitive strain (*SI Appendix*, Fig. S7). By varying the RIF concentration, we could thus tune the selective advantage of the resistant versus sensitive strain. After approx. 40 h of incubation in pure LB medium in the microfluidic device, which resulted in the establishment of fluorescent sectors, we changed the medium to 2 µg/mL RIF in LB (“low RIF”), and then after another 50 h to 3.5 µg/mL RIF (“high RIF”), just below the MIC of RIF-sensitive strain. Fig. 4*C* shows that the fraction of the RIF-sensitive green fluorescent strain decreased in all wells during the exposure to RIF, with a more pronounced reduction in wells with flat bottoms, and corrugated bottoms with wider undulations. Replacing RIF with pure LB medium led to the successful reestablishment of the sensitive strain in corrugated wells with T<100 μm (Fig. 4 *C* and *D* and Movie S2). However, reestablishment occurred rarely in flat-bottom wells or those with T=100 μm. Fig. 4*E* quantifies this effect by plotting the ratio of the sensitive strain fractions at two time points: after (t = 182 h) and before (t = 41 h) the exposure to RIF, for different well types. A similar trend is visible in the number of RIF-sensitive sectors (*SI Appendix*, Figs. S8 and S9). We also obtained qualitatively similar results using a protocol with a single RIF step-up of 4 to 4.5 μg/mL (*SI Appendix*, Fig. S10).

The rate with which a fitter sector expands can be related to the relative fitness of the sensitive versus the resistant strain (**SI Appendix*, Methods*). By fitting the model to the experimental time-series data for flat-bottom wells, we determined the relative fitness $W_{S/R}\approx 0.2$ at high RIF (*SI Appendix*, Fig. S11). Next, we ran the no-adhesion version of the computer model, using initial clonal fractions obtained at the end of the low-RIF phase of the experiment from Fig. 4*C*, the relative fitness at high RIF $W_{S/R}=0.2$ and undulation amplitudes as in Fig. 4*E*. The model correctly reproduced the mean sensitive clonal fraction at the end of the experiment (Fig. 4*F*). Interestingly, repeating the same procedure for the model with adhesion to the surface strong enough to significantly affect the velocity field of the growing biofilm yielded a much worse fit (Fig. 4*F*). This does not mean that cells did not adhere to the walls but only that surface corrugations were more important than adhesion for the outcome of our experiment.

## Discussion

We have shown that biofilm population dynamics depends on the shape of the surface on which the biofilm grows and that surface corrugations of appropriate period and amplitude can prevent clonal subpopulations from invading each other. This reduces genetic drift, enabling neutral variants to coexist, while also diminishing the impact of selection, preventing fitter variants from dominating over less-fit clones. These effects arise from the interplay of bacteria–surface interactions that influence cell orientation and the velocity field in the biofilm. Importantly, the depth of the corrugations required to see these effects is only a small fraction of the biofilm’s thickness, and the corrugations do not physically separate different regions of the biofilm.

Our model system exhibits significant differences compared to bacterial range expansion experiments on agar plates (21, 24, 25, 43, 50). Clonal sectors emerge despite uniform growth throughout the entire biofilm volume, whereas on agar plates, a thin growing layer is necessary for clonal segregation to occur. Additionally, in our system, the roughness of the biofilm’s top surface has no effect on its population dynamics, unlike in colonies on agar where the roughness of the leading edge significantly affects genetic drift (20). This is because in our system, it is the growth at the bottom, not at the leading edge (top surface), that drives the dynamics of clonal sectors. Interestingly, static, i.e., time-independent undulations of the bottom surface have the opposite effect compared to dynamically changing undulations of the expanding frontier of a growing colony (16, 43): Genetic drift is reduced rather than enhanced by static roughness.

Computer simulations from Fig. 4 *B* and *F* show that surface adhesion could have a profound effect on selection, reducing the influence of growth rate differences on the probability of establishment of fitter variants. Since in our model, fitter clones must first expand close to the bottom surface to dominate the biofilm, we conclude that adhesion could partially counterbalance the effect of increased growth rate and hinder expansion. In our experiment, this effect does not seem to be very strong, perhaps because intercellular adhesion is stronger than adhesion to the walls, but it certainly plays a role in flat-bottom wells, sometimes preventing a full takeover of the fitter clone. Nevertheless, we hypothesize that mutants with reduced adhesion could gain an additional, growth-independent selective advantage, similarly to what happens in bacterial colonies (51). However, the advantage might be short lived: Since adhesion is essential for biofilm establishment on surfaces, less-adherent variants could eventually cause the biofilm to detach. Consequently, conducting an experiment with a less adherent strain of bacteria would not be possible in our setup as it would result in biofilms falling out of the wells.

Surface adhesion is likely responsible for the observed persistence of neutral sectors in some flat-bottomed wells. In a well-mixed growing population whose size remains approximately constant due to the continuous removal of surplus population, one clone would eventually always reach fixation (52). However, in our system, adhesion may prevent fixation, particularly if certain cells (e.g., older ones) adhere stronger than others. Such cells may persist in the well for an extended period, leading to the formation of long-lasting sectors by their progeny.

A key mechanism driving clonal expansion on flat surfaces and within sine-like pockets is the horizontal component of the velocity field near the surface (Fig. 2). In the computer model (Fig. 3), this component arises due to “buckling,” i.e., pressure-driven bending of chains of bacteria experiencing growth-induced compression (28, 30, 53, 54). This is supported by the lack of lateral motion of cells in simulations in which the height of the biofilm has been made much smaller, reducing mechanical stress and making cells more aligned with the flow, and the restoration of lateral movement upon increasing the friction coefficient (Movies S3–S5). In the actual experiment, buckling manifests as bends in fluorescent sectors, particularly noticeable near the bottom. Notably, buckling is essential for the velocity flow to acquire a horizontal component, causing cells at the bottom of the well to orient themselves perpendicularly to the surface. For corrugated surfaces, the onset of lateral buckling occurs earlier, i.e., for smaller compression forces, because the curvature of such surfaces prevents cells from aligning into linear chains. This explains why corrugated surfaces hinder mixing: Earlier buckling (more pronounced for surface undulations of higher amplitude) results in a faster transition from horizontal to vertical biofilm flow.

Since buckling is affected by adhesion and friction not only with the bottom and side walls but also the glass and PDMS surfaces, we expect that our results would be quantitatively—but not qualitatively—different, if we used thicker wells to accommodate more cell layers. Creating a fully three-dimensional biofilm would reduce the role of such boundary effects. Patterning the surface in two directions would be necessary to limit clonal expansion under such conditions.

Our quantitative results, the number of sectors and their sizes and the fraction of sensitive/resistant cells in different types of wells, depend not only on the geometry of the well but also factors such as the initial fraction of cells of each type, their location in the well, and when individual cells resume growth after inoculation (lag time). We could not detect any significant heterogeneities in the lag time, but we observed different initial cell densities in different wells (*SI Appendix*, Fig. S3 *C* and *D*). However, the initial density was always high enough to only minimally influence the number of sectors formed (*SI Appendix*, *Methods* and Fig. S3 *E* and *F*).

Many population genetics models (49, 55–59) rely on compartmentalization to influence population dynamics, which may not be applicable to growing biofilms. In contrast, our work shows how the physics of the biofilm can lead to effective spatial separation, which helps to maintain genetic diversity. We speculate that our findings generalize to naturally occurring biofilms that are relatively thin (a few hundred µm) to enable growth at the bottom. If the biofilm is thick enough to prohibit cell division at the bottom but its height remains limited due to mechanical shearing or flow, the shape of the surface it adheres to should become less important. Nevertheless, this might change in the presence of particles such as microplastics attaching to the biofilm and creating new adhesion points for cells.

Our results suggest that bacterial population dynamics in the biofilm can be controlled by manipulating the surface geometry. This can be used to restrict the expansion of undesired genetic variants; we demonstrate this specifically for an antibiotic-resistant mutant. Sub-MIC transient antibiotic exposure is not uncommon and may lead to the emergence of resistance (47, 60). However, further research is required to see whether surface patterning could be used for medical devices such as catheters or implants, which are prone to biofilm invasion (37). Similar strategies could also be utilized to stabilize engineered bacterial communities (61), in particular for the use in biosensing (62) or bioremediation, in which different bacterial ecotypes must often coexist together (63), and mutations in synthetic gene networks that occur in such communities must be suppressed.

## Materials and Methods

### Microfluidic Device.

We fabricated a two-layer device made of PDMS attached to a glass slide (*SI Appendix*, Fig. S1), with 240 microwells on both sides of a 500 µm wide and 87 µm deep channel. Each well measured 100 × 100 × 7 µm [width (X) × height (Y) × depth (Z)]. The device contained 20 replicates of each of eight sine wave/amplitude combinations and 80 flat-bottomed wells. To fabricate the device, we utilized soft lithography, following well-established protocols (**SI Appendix*, Methods*). A photomask designed in AutoCAD (Autodesk) and printed by MicroLitho, UK, was used to expose a layer of negative photoresist on a silicon wafer. After developing, the negative mold was covered with PDMS (Sylgard, Dow Corning) mixed at a 1:10 ratio of curing agent to monomer and baked at 75 °C for at least 4 h. The resulting PDMS device was peeled off, oxygen-plasma-treated, and bonded to a plasma-treated 1 mm thick glass slide. After inserting PTFE tubing (Bola Bohlender, Germany, I.D. = 0.5 mm, O.D. = 1.0 mm), the device was connected to syringe pumps PHD2000 (Harvard Apparatus, USA) or (in some experiments) SyringeONE Programmable Syringe Pump (Darwin Microfluidics). We used plastic syringes with appropriate media (bacterial culture, LB, LB + rifampicin), depending on the type of experiment. Prior to use, all devices were flushed with 5% NaOH in 70% ethanol to sterilize the device, remove air, and enhance bacterial adhesion.

### Bacterial Strains.

We used the *E. coli* strain 83972 (64) and its fluorescent derivatives for all experiments, with either mKate (red) or GFP (green) being constitutively expressed from the bacterial chromosome. For the experiments with a RIF-resistant strain, we used a variant of the red fluorescent strain with resistance conferred by a single-point mutation of the *rpoB* gene. The presence of the fluorescent reporter did not reduce fitness compared to the ancestral strain, but rifampicin resistance decreased fitness by about 20% (*SI Appendix*, Fig. S7). All strains easily adhered to surfaces. To confirm that bacteria in the wells showed hallmarks of biofilm formation such as matrix production, we stained them with bromophenol blue (curli) and EbbaBiolight 680 (amyloids and certain glucans) (*SI Appendix*, Fig. S12). All genetic modifications are detailed in **SI Appendix*, Methods*.

### Biofilm Growth and Imaging.

All experiments were performed at room temperature (25 ± 0.5 °C). We inoculated the microfluidic device through the attached PTFE tubing using dense bacterial cultures that were grown overnight in LB (Miller) broth (Carl Roth, Germany) at 37 °C/180 rpm, mixed in desired ratios (1:1 or 1:10 as established by OD_600_ measurements), and centrifuged to increase the cell concentration. After allowing bacteria to settle in the wells for approx. 30 min, we swapped the syringe with the bacteria to a new one filled with LB broth and initiated the flow. We used variable-flow rate protocols (**SI Appendix*, Methods*) to reduce clogging and biofilm growth in the main channel. For the experiments in Fig. 4 and *SI Appendix*, Fig. S10, we replaced the LB medium with LB + RIF, and later switched back to LB, as described in the main text.

### Microscopy and Image Analysis.

Images were acquired on two fully automated Nikon Ti2-Eclipse epifluorescent microscopes equipped with automated XY stages, the Perfect Focus System, and GFP and mCherry fluorescent filters. The microscopes were controlled by MicroManager (65). Depending on the experiment, we used either 20× or 40× long-working distance Nikon objectives. Custom-written Python and Mathematica® code was used to load and process raw TIFF images outputted by MicroManager and to analyze and plot the data. Image registration was used to compensate for small movements of the device during biofilm imaging for the sensitive optical flow method (*SI Appendix*, Fig. S13). See **SI Appendix*, Methods* for details.

### Computer Model.

Computer simulations were conducted on the Edinburgh compute cluster at SoPA. We adapted the model previously reported (20), which involved representing bacteria as growing spherocylinders, with their movement confined to the XY plane, and interacting mechanically through Hertzian-like repulsion. The dynamics was overdamped with Stokes-like friction proportional to the velocity of the moving cell. We did not model nutrient diffusion and assumed that all cells of the same type grew at the same rate, regardless of their location in the well. We modeled repulsion from the walls of the well as a force that increased linearly with the overlap between the cell and the wall, with a stiffness constant $10^{7}$ pN/μm sufficiently large to prevent the overlap to increase beyond a fraction of a µm. Adhesion between bacteria and walls was represented using elastic springs (spring constant $k=10^{6}$ pN/µm), connecting the center line of the cell to the closest point of first contact on the wall. The springs broke when extended by more than $\epsilon_{\max}$ = 5% or 20% of their initial length.

### Use of AI Tools.

ChatGPT was used to refine the text and improve its flow, although no ChatGPT-generated sentences were copied verbatim into the manuscript. The manuscript was read and approved by all authors.

## Supplementary Material

Appendix 01 (PDF)

Movie S1. Biofilm growth in selected wells (as in Fig. 1) from inoculation (t=0) until t=144 h.

Movie S2. Biofilm growth in 4 wells of different type (as in Fig. 4): flat, (T,A)=(10,1.7), (T,A)=(20,5.1), and (T,A)=(50,8.7) μm, from inoculation (t=0) until t=182 h.

Movie S3. Simulated biofilm in a flat-bottom 80x70 μm well, for the same parameters as simulations presented in Fig. 4A.

Movie S4. Simulated biofilm in a flat-bottom 80x30 μm well, for the same parameters as Movie S3.

Movie S5. Simulated biofilm in a flat-bottom 80x30 μm well, for the same parameters as Movie S3, with the exception of the friction coefficient being 20x larger.

## Data Availability

All code (C++, Jupyter notebooks, and Mathematica notebooks), processed image data, and simulation results are available from a GitHub repository (https://github.com/Dioscuri-Centre/biofilms_on_corrugated_surfaces) (66). Due to large file sizes (several TBs), raw image data have not been uploaded. Access to such data will be provided on request.
