# Supplementary material for: Substrate geometry affects population dynamics in a bacterial biofilm
Source: Proc Natl Acad Sci U S A. 2024 Apr 15;121(17):e2315361121. doi: 10.1073/pnas.2315361121 (PMC11047097; doi:10.1073/pnas.2315361121)
Supplement: Supplementary file 1 — Appendix 01 (PDF) [file pnas.2315361121.sapp.pdf]

PNAS

Supporting Information for

Substrate geometry affects population dynamics in a bacterial biofilm

Witold Postek*, Klaudia Staśkiewicz*, Elin Lilja, Bartłomiej Wacław

Corresponding authors: Witold Postek, Bartłomiej Wacław
Emails: wipostek@gmail.com; bwaclaw@ichf.edu.pl

*= these authors have contributed equally to this work

This PDF file includes:

Supporting text
Figures S1 to S13
Table S1
Legends for Movies S1 to S5
SI References

Other supporting materials for this manuscript include the following:

Movies S1 to S5

Supporting Information Text

Microfluidic device architecture

The microfluidic chip used in our study was a two-layer device consisting of a PDMS mold attached to a 1 mm-thick glass slide (SI Fig. S1). The device featured one inlet and two outlets equipped with pillar-based filters to prevent inflowing debris. The main channel was 500 µm wide. Micro-wells for culturing biofilms extended perpendicularly from the main channel; each was 100 µm wide (parallel to the main channel) and 100 µm deep (perpendicularly to the long axis of the main channel). The final height of the main channels and the wells obtained through soft lithography (see below) was 87 and 7 µm, respectively, so that only a few layers of cells could fit into each well, whereas the much taller main channel allowed for rapid nutrient medium flow. The 7 µm well thickness facilitated optical imaging while at the same time ensuring that most bacteria interacted with other bacteria in the bulk of the biofilm rather than with the top (PDMS) or bottom (glass) surfaces. The biofilms were continuously trimmed by the flow in the main channel, ensuring a steady supply of nutrients to the deepest layers of the biofilm.

Each microfluidic device had 240 micro-wells, with 120 on each side of the main channel. The bottom of the well, which faced away from the main channel, was designed to be either flat or undulated. The shape of the undulations was a sine function of different periods and amplitudes (all dimensions in µm): $(T, A) = ((100,9.5), (100,5.1), (50,8.7), (50,4.6), (20,5.1), (20,3.3), (10,1.7), (10,1.3))$. Due to the limitations of soft lithography and mask resolution, it was not possible to have the same amplitude A for all periods T. The reported amplitudes are actual amplitudes obtained from microphotographs of the device. Each device contained 20 replicates of each sine wave / amplitude combination, as well as 80 flat-bottomed wells. The CAD design of the device is available on GitHub (1).

Soft lithography

To create a negative of the microfluidic device, we followed well-established photolithography protocols (2). First, we designed a photomask in AutoCAD (AutoDesk) and had this mask printed by an external company (MicroLitho, UK). We then covered a 3-inch silicon wafer (Microchemicals) with an SU-8 photoresist (Kayaku Advanced Materials) using a spin coater (Laurell, USA). After a soft bake on a programmable hot plate (4 minutes at 95°C), we exposed the wafer through the photomask representing the micro-wells layer, using a MJB4 mask aligner (SÜSS MicroTec). The second layer of SU-8 was then spun on the wafer, and the wafer was again soft-baked (5 minutes 65°C, 20 minutes 95°C). Edge bead-removal procedure was applied using the spin coater by covering the edge of the spinning wafer with photoresist developer mr-600 (Micro Resist Technology, Germany), deposited through a syringe, to unravel the alignment marks from the first layer. The wafer then underwent another soft bake (5 minutes 65°C, 20 minutes 95°C). Next, the wafer was illuminated through the photomask representing the main channel, and a post-exposure bake (5 minutes 65°C, 10 minutes 95°C) was performed. The specific spinning times, speeds, soft bake/hard bake times, and illumination parameters were obtained from the SU-8 manufacturer's (Kayaku) protocols. The wafer was developed with mr-600 developer (Micro Resist Technology, Germany) according to the manufacturer's protocol. After the development, the wafer was hard baked at 150°C for 30 minutes.

Microfluidic device casting

The wafer with the photo-resist negative of the device was covered with PDMS (Sylgard, Dow Corning) mixed at a 1:10 ratio of curing agent to monomer, and baked at 75°C for at least four hours. The cured PDMS was peeled off the wafer and inlet and outlet holes were created using a 1 mm-diameter biopsy puncher (Kai Medical). The PDMS device was activated with oxygen plasma alongside a glass slide in a plasma cleaner (Harrick Plasma, USA) for 60 seconds. Following this, the PMDS mold was gently placed on the glass slide and lightly pressed with a pair of metal tweezers to ensure proper bonding of the PDMS to the glass.

Bacterial strains

E. coli 83972 (DSM number 103539) was obtained from DSMZ GmbH. The red fluorescent mKate marker (under the control of the constitutive promoter *PtetO1*) was introduced into 83972 using plasmid mediated gene replacement (3, 4) replacing the *galK* gene. The strain used to amplify the mKate marker with the *PtetO1* promoter from was a gift from Meriem El Karoui (5). The green fluorescent GFP marker under the control of the constitutive *PA1* promoter was introduced into *E. coli* 83972 using plasmid mediated gene replacement, replacing the *galK* gene. The GFP marker with the *PA1* promoter was amplified from plasmid pGRG36-Kn_PA1-GFP (6). Plasmid pGRG36-Kn-PA1-GFP was a gift from Frank Rosenzweig (Addgene plasmid # 79088 ; http://n2t.net/addgene:79088 ; RRID:Addgene_79088). The primers used to amplify the upstream and downstream regions surrounding the *galK* gene of 83972, as well as the primers used to amplify the mKate and the GFP markers can be found in Table S1 below. Crossover PCR was used to anneal the 83972 homologous regions with the mKate and GFP markers, and these constructs were then inserted by restriction digestion and ligation into the plasmid pTOF24 (4) used for the gene replacement.

A rifampicin (RIF) resistant version of 83972 with the mKate marker was generated by plating the strain on LB agar plates supplemented with 100 µg/ml rifampicin, and randomly picking a resistant colony. To identify the genetic basis for the resistance we sequenced the strain using a MinION (Oxford Nanopore) sequencing device with a R9.4.1 flow cell and the whole genome library prepared with the Rapid Barcoding Kit SQK-RBK110.96. We identified a single nucleotide polymorphism in the *rpoB* gene conferring the amino acid change H526Y. All genomic data for this study have been deposited in the European Nucleotide Archive (ENA) at EMBL-EBI under accession number PRJEB72646 (https://www.ebi.ac.uk/ena/browser/view/PRJEB72646)

1.galK_up_fwd	AAA AAC TGC AGA CAC TGG TTA GCC GTT GTA C
2.galK_up_rev	ATA GGG ACT CGA TTTC TTA CAC TCC GCA TTC
3.mKate_gal_fwd	GGA GTG TAA GAA TCG AGT CCC TAT CAG TGA
4.mKate_gal_rev	CGG GAG TTT CGT TTA TCT GTG CCC CAG TTT
5.galK_down_fwd	GGG CAC AGA TAA ACG AAA CTC CCG CAC TGG
6.galK_down_rev	AAA AAG TCG ACT GAT CGC CAT CAT CTG AAC T
7.Gal_up_fwd	AAA AAC TGC AGT GAC GAT CGT TCT GGT TCA C
8.Gal_PA1_up_rev	TGA TAA CCG CTA CGG AAG AGC TGG TGC CTG
9.PA1_gal_fwd	CCA GCT CTT CCG TAG CGG TTA TCA AAA AGA
10.GFPt7_gal_rev	GGA GTG TAA GAA TCA GCA AAA AAC CCC TCA
11.Gal_GFPt7_down_fwd	GTT TTT TGC TGA TTC TTA CAC TCC GGA TTC
12.Gal_down_rev	AAA AAG TCG ACA CAC TGG TTA GCC GTT GTA C

Table S1. Primers used to amplify the upstream (1,2) and downstream (5,6) regions of the galK gene of 83972, with overlap to PtetO1_mKate, primers used to amplify mKate with the PtetO1 promoter (3,4), primers to amplify the upstream (7,8) and downstream (11,12) regions of the galK gene of 83972, with overlap to PA1_GFP, and primers to amplify GFP with the PA1 promoter (9,10).

Bacterial cultures

Single colonies were grown from frozen stocks on Luria broth (LB) agar plates at 37°C for 24h. Liquid cultures were prepared by inoculating 10 mL LB (Miller, Carl Roth, Germany) using a

single colony, and incubated overnight in a shaken incubator (37°C at 180 rpm). After the overnight incubation, the cultures were mixed in a 1:1 ratio (Figure 1, GFP:RIF^S to mKate:RIF^S) or 10:1 ratio (Figures 4 and S10, GFP:RIF^S to mKate:RIF^R), according to their optical densities (OD600), centrifuged at 4000 rpm for 2 min, and re-suspended in 1 mL LB to concentrate them approximately ten-fold.

Growth media and flow control
We used LB Broth (Miller) sterilized by autoclaving at 121°C for 15 min, and supplemented with rifampicin (RIF) (Merck KGaA, Germany, concentrations as in the main text) for the experiments in Figs. 4 and S10. The medium was delivered by syringe pumps using plastic syringes (BD, USA). The syringes were connected to the microfluidic devices with PTFE tubing (Bola Bohlender, Germany, I.D. = 0.5 mm, O.D. = 1.0 mm), with identical lengths for both outlets to ensure equal hydraulic resistance. 0.5 mm OD needles were used to connect syringes to the tubing. Syringe pump PHD2000 (Harvard Apparatus, USA) was used for experiments in Fig. 2. Experiments from Figs. 1, 4 and S10 used a SyringeONE Programmable Syringe Pump (Darwin Microfluidics).

Biofilm experiments
The microfluidic device was flushed with a solution of 5% sodium hydroxide (NaOH, Carl Roth, Germany) in 70% ethanol using a syringe mounted in a syringe pump, at a rate of 0.2 ml/h for 8 min. Afterwards, the device was flushed with 70% ethanol, followed by LB medium. A dense bacterial suspension was then introduced into the device using a syringe pump at a flow rate of 3 ml/h. When bacteria showed up in the main channel, the flow rate was reduced to 200 μL/h and periodically turned on and off for about 30 min, to make the bacteria attach, while continuously imaging the wells until they contained hundreds of cells/well. The bacterial syringe was then replaced with an LB syringe. The flow rate was set to 50 μL/h for 400 μL (8 h), followed by an alternating fast/slow flow of 15 ml/h for 10 μL and 50 μl/h for 16 μL to reduce clogging of the main channel in the 180 h-long experiment. For the experiments in Figs. 4 and S10, the medium was replaced with LB+RIF and then LB again, as described in the main text. To confirm presence of typical biofilm hallmarks such as amyloids or glucans, we chose two fluorescent dyes: bromophenol blue (15) (SI Fig. S12, left), which binds to the amyloid curli, and EbbaBiolight 680 (EbbaBiotech AB, Sweden), (SI Fig. S12, right) optimized to stain amyloids and certain glucans. In the case of bromophenol blue, the biofilms were stained by 100 μM of the dye in LB, for 10 minutes, and then washed with PBS for 10 minutes, at a flow rate of 2 ml/h. In the case of the EbbaBiolight 680 stain, the biofilms were first washed with 1 ml of PBS at a flow rate of 10 ml/h, then stained with a 1:1000-fold dilution in PBS for 2 hours, initially with 0.5 ml at a rate of 10 ml/h, then for the remaining time with 50 μl/h, and then finally washed with PBS again at 10 ml/h for 30 minutes.
We ran all experiments in an incubator set to 25°C; this helped to limit biofilm growth in the main channel. Experiments were performed in three biological replicates (Fig. 1), one replicate (Fig. 4), and three replicates (Fig. S10).
While we always mixed the strains in the same 1:10 resistant:sensitive ratio for the experiments in Figs. 4 and SI Fig. S10, the actual ratio of bacteria that adhered to the wells of the microfluidic chip was different. This was because bacteria collided and formed clumps in the syringe used for inoculation, and these collisions occurred more often for the more abundant sensitive strain. Since only single cells attached in the wells as evidenced by imaging, and clumping enriched the cultures in the less abundant, resistant strain, the ratio of resistant:sensitive strain was higher than 1:10 in the wells.

Microscopy
Images were acquired using two fully automated Nikon Eclipse Ti2-E epi-fluorescent microscopes with automated XY stages and the Perfect Focus System, and controlled by MicroManager (7). One microscope used an ORCA-spark Digital CMOS camera C11440-36U (Hamamatsu, Japan), the other one an Andor Zyla 4.2 SCMOS camera (Oxford Instruments,

UK). Depending on the experiment, we used two different objectives (20x NA 0.45 and 40x NA 0.6). To acquire fluorescent images of mKate and GFP strains, we used filters with excitation/emission wavelengths of 532 – 554 nm / 573 – 613 nm, and 457.5 – 487.5 nm / 502.5 – 537.5 nm. To acquire images of bromophenol blue and EbbaBiolight 680 stains, we used filters with excitation/emission wavelengths 532 – 554 nm / 573 – 613 nm and 542 – 582 nm / 604-644 nm, respectively.

Image and data analysis

To analyze the data, we utilized custom Python and Mathematica® code to load and process the TIFF images generated by MicroManager, and raw data files from the plate reader control software. The code allowed us to perform the necessary data analysis and create plots for visualization. The code is available on GitHub as Jupyter and Mathematica notebooks (1).

Figure 1D and SI Fig. S2. To calculate the probability distribution of finding a sector boundary at position x, we first extracted the intensity profiles in red (mKate) and green (GFP) channels along a horizontal line (x coordinate) parallel to the well's bottom (just above the undulations, or at the bottom for flat wells). Pixel intensities in both channels were rescaled by the average values from the entire acquisition (all wells and time points) to account for differences in overall red/green fluorescence. We then calculated the quantity $p(x) = -f_1'(x)f_2'(x)$, where $f_1(x), f_2(x)$ were fluorescence intensities at position x, and primes denote derivatives with respect to x. This quantity is maximized at sector boundaries, where fluorescence changes rapidly in both channels. We called a pixel to belong to sector boundary whenever $p(x)$ was larger than 10% of the maximum value of $p(x)$. Note that this procedure only identified regions in x in which sector boundaries were likely and was not suitable for detecting precise positions of the boundaries. See the next paragraph for an alternative approach which is however more sensitive to image noise and non-even fluorescence within the sectors.

SI Figure S3ABG. Fluorescence intensity profiles along the horizontal line near the bottom (as for Fig. 1) were rescaled by average values as described above and then logarithmized as follows: $I_{\text{rescaled}} = \ln(I_{\text{raw}} - I_{\min} + 1) / \ln(I_{\max} - I_{\min})$. The 1d array obtained in this way was convolved with a top hat function of width 10 pixels to reduce noise. Next, we detected the boundaries between red and green sectors based on pixel intensity in each channel. A pixel was classified as belonging to a "red" sector if its intensity was higher in the red channel than in the green channel, and vice-versa for the "green" sector. Using sectors boundaries determined in this way, we calculated the number of sectors and their sizes for each well and time point. We then averaged the sector sizes over all wells of the same type.

Figure 2. We imaged the biofilm every second for 3 minutes using the 40x objective. The biofilm grew only minimally during this time, and individual cells moved less than a pixel per time frame. Inspired by Refs. (8, 9), we determined the velocity field $\vec{v}(x,y)$ from subtle changes in pixel brightness caused by the biofilm's local motion. This method did not require tracer particles or feature detection within the biofilm. Briefly, we assumed that the flow in the biofilm caused the pixel intensity field $I(x,y,t)$ to evolve during a short time interval dt as follows: $I(x,y,t+dt) = I(x,y,t) - dt\, \vec{v}(x,y) \cdot \vec{\nabla} I(x,y,t)$, where $\vec{\nabla}$ represents the (discrete) gradient operator. This linear set of equations (one for each pixel) could be solved numerically for $\vec{v}(x,y)$ using pixel intensities at two time points. Since the system of equations was underdetermined, we binned the image 32x in each direction and solved for the two components of the average velocity field within each 32x32 block of pixels using the least squares method. Additionally, we automatically selected the time separation dt for each bin that yields the most accurate estimate for $v(x,y)$ at that location without violating the assumption of small changes ($v\, dt < 0.1$ bin size). We used the same approach for data presented in SI Figs. S5, S6, except that we used the 20x objective and imaged for 1 min.

Figure 4 and S10. We first followed the same approach as for Fig. 1. To find the fraction of sensitive (green) strain, we calculated the proportion of "green" pixels ($I_{\mathrm{G,rescaled}} > I_{\mathrm{R,rescaled}}$) along the line of pixels. This method worked reliably once green and red strains separated into sectors, which is the reason for the apparent decrease of the mean sensitive fraction in Fig. 4C during the first part of the experiment (no RIF), despite the red strain having a small fitness disadvantage. We also confirmed that this method gave qualitatively similar results to the manual counting of sectors (SI Fig. S8).

The initial distribution of bacteria (SI Fig. S3CD)
We determined the positions of cells in all flat-bottomed wells in the experiment from Fig. 1, at $t = 30$ min from the end of inoculation. To find the cells, we searched for bright spots in fluorescent images using a custom-made Python script. The number of detected cells was within 20% of the number obtained by manual counting in a sample of 5 wells. SI Figure S3C shows that the distribution of cells as a function of the distance d from the bottom, is consistent with a uniform distribution. To confirm this, we tested each well against the uniform distribution using the Kolmogorov-Smirnov test. SI Figure. S3D shows that all wells have p-values above 0.05, and the distribution of p-values is quite uniform, as would be expected if the null hypothesis (uniform distribution of cells) was true.
The average initial number of cells per well is $N_{\mathrm{initial}} = 294 \pm 8$ (minimum = 188, maximum = 414), and the ratio of red to green cells is 1.08 ± 0.01, very close to the assumed ratio 1:1. To determine the effect of N_{initial} on the number of sectors formed, we run simulations starting from different initial densities of cells, each cell being assigned a different type. SI Figure S3E-F shows the number of sectors after 72 h of a simulated experiment, for flat-bottomed and corrugated ($A = 1.3\ \mu\mathrm{m}, T = 10\ \mu\mathrm{m}$) wells. The final number of sectors depends very little on N_{initial}, for $N_{\mathrm{initial}} > 200$, which is the range of initial cell densities observed in the experiment.

Image registration for velocity field time-lapse videos (SI Fig. S13)
We monitored positions of small defects in the PDMS which appeared as bright or dark spots in the brightfield channel. We selected 5-10 such spots per field of view (FOV), uniformly distributed around the wells in that FOV, and fitted a Gaussian to each of them, for all time points. We then calculated the average velocity $\vec{v}(t)$ for each spot by fitting a linear function $\vec{r} = \vec{r_0} + \vec{v} \cdot t$ to the position of the centre of mass of the Gaussian, and averaged the velocities over all spots from the same FOV to obtain the mean velocity of the PDMS device in that FOV. SI Figure S13 shows that the PDMS velocities in videos used to generate Fig. 2 are very small (typically <1 μm/h) compared to the velocities in the biofilm (10's of μm/h) except for regions very close to the bottom.
We applied the same procedure of image registration to data sets from Fig. 1 and subtracted the detected PDMS velocity (usually less than 1 μm/h in each direction) from the velocity field. In a few FOV in which we detected movement with velocities >1 μm/h, we visually checked that the subtraction fully compensated for the movement of the PDMS device.

Biofilm growth rate and its dependence on biofilm age (SI Fig. S5BCD)
While performing experiments for Fig. 1, we measured velocity fields at six time points: 20 h, 29 h, 69 h, 146 h, 167 h, and 192 h. SI Figure S5 shows the vertical component $v_y(d)$ of the velocity field versus the distance d from the bottom, for 6 different FOVs with 4 flat-bottom wells per FOV, and two time points $t = 20$ h (panel B) and $t = 192$ h (panel C). Panel D shows the growth rate obtained from a linear fit to these data, for all six time points. The plots show that

- $v_y(d)$ increases approximately linearly with d in most of the wells, for both the young and old biofilm, confirming that the local growth rate depends very little on d
- the growth rate decreases in time, possibly due to the biofilm ageing
- the growth rate is higher for wells closer to the nutrient inlet and decreases towards the outlet. This could be caused by nutrient depletion/waste accumulation near the sides of the main channel to which the wells open.

Doubling time of bacteria in the biofilm before rifampicin (Figs. 4 and S10)

For the biofilm from Fig. 4, we tracked the movement of easy-to-distinguish features (bright spots and swirls) in fluorescence images of biofilms growing in flat-bottomed wells during the first phase of the experiment presented in Fig. 4 (pure LB, no RIF). If $d_t, d_{t+\Delta t}$ denote the distance of a feature from the bottom at times t and $t + \Delta t$, then the growth rate α can be calculated as

$$\alpha = \frac{\ln\left(\frac{d_{t+\Delta t}}{d_t}\right)}{\Delta t}.$$

We calculated α for all flat-bottomed wells, using 2-3 traceable features per well. We obtained the average value $\alpha = 0.22 +/- 0.01$ h^{-1}, corresponding to the doubling time ≈3 h. We did not observe any significant correlation between α and d_t of the feature (Pearson correlation coefficient $r = 0.10$, p-value 0.40), which confirmed that growth did not depend on the depth (distance from the well opening) in the biofilm.

For the biofilms from SI Fig. S10, we used the optical flow method described above. The average growth rate from plots in SI Fig. S6 was $\alpha = 0.35 \pm 0.03$ h^{-1}.

In both cases, the growth rate in the biofilm was significantly lower than in the liquid culture (SI Fig. S7) at the same temperature. It was also lower than the growth rate in a younger biofilm from Fig. 2.

In contrast to previous work (10), where mechanical confinement to a monolayer of cells has been suggested as a possible explanation of reduced growth rate, in our experiment the movement of cells is not constrained enough to cause axial compression, which could affect the growth rate (11). We hypothesize that cells in the biofilm switch to a slower-growing phenotype, perhaps as the effect of quorum-sensing (12), or that a nutrient-depleted boundary layer develops close to the wells opening, despite a fast flow in the center of the main channel.

Relative fitness from sector expansion (SI Fig. S11)

We used data from Fig. 4C to obtain the relative fitness $W_{S/R}$ of the sensitive green strain compared to the resistant red strain at high RIF. We first selected flat-bottomed wells in which the initial fraction of resistant (red) cells was between 0.1 and 0.4 at the beginning of the 45 h-long high-RIF phase from Fig. 4C. The [0.1, 0.4] range was chosen because the sector detection algorithm performed well in this range. SI Figure S11A shows plots of the resistant fraction versus time for the selected wells. We then ran the computer model in flat-bottomed wells for different resistant initial fractions and different relative fitness values $W_{S/R}$. We assumed the doubling time of 3 h (see the preceding section) and the same duration of the simulation as the high-RIF phase. We selected runs for which the initial fraction was within ± 0.01 of the experimentally determined values, thus the simulated curves (SI Fig. S11B) had the same initial distribution of resistant fractions as the experimental curves. Finally, we compared the theoretical and simulated average resistant fraction versus time curves obtained for different $W_{S/R}$, and determined that the best fit was given by the model assuming $W_{S/R} = 0.2$ and no adhesion.

Relative fitness from liquid culture growth (SI Fig. S7)

We used the method from previous work (13) to determine the growth rate of strains mKate:RIF^R and GFP:RIF^S at different concentrations of RIF. Briefly, we incubated bacteria in LB with different concentrations of RIF (0, 2, 4, 5, 6, and 8 µg/ml) in a 96 well micro-plate (200 µl/well) in a plate reader, starting from two different initial cell densities, N_0 in rows A-D and $N_0/10$ in rows E-H. The N_0 dilution was an exponentially growing culture of OD600 ≈ 0.1 diluted 1:200 into LB. We used one column of the 96 well plate for each RIF concentration. The plate reader (BMG LABTECH FLUOstar Optima) was set to incubate the plate at 25 °C

in a room with ambient temperature below 18 °C for enhanced temperature stability, with orbital shaking at 200 rpm for 10 s prior to OD measurement.

We measured the optical density (OD) of each culture every 2 min to obtain growth curves for approx. six days of incubation time. The exponential growth rate was then determined from the time shift between the growth curves for initial bacterial concentrations N_0 and $N_0/10$.

SI Figure S7A shows that the two fluorescently-marked strains have almost identical growth rates in the absence of RIF. The sensitive green strain grows about 20% faster without RIF ($W_{S/R} = 1.22$), but at 3.5 μg/ml used in experiments from Fig. 4 its relative fitness is $W_{S/R} \approx 0.5$ compared to the red resistant strain. This differs from the estimate from the previous section ($W_{S/R} \approx 0.2$) based on fitting the computer model to the sector expansion experiment in the biofilm. The difference could be due to different conditions in mature biofilms (slower growth) or RIF degradation in the plate (in contrast to the microfluidic device, there is no influx of fresh RIF in the plate).

The MIC of the sensitive strain is about 5 μg/ml in the plate.

We also compared the growth rates of sensitive variants mKate:RIF^S and GFP:RIF^S using the same assay. SI Figure S7B shows that the strains have the same growth rate in pure LB, in agreement with the competition assay (see below).

Relative fitness from a competition assay in bulk cultures

The relative fitness of the mKate-labeled 83972 strain as compared to the GFP-labelled strain was determined by setting up a competition assay of the strains against each other in LB broth, shaking at room temperature (25 °C). Overnight cultures of both strains were mixed in a 1:1 ratio, diluted 1000-fold into four replicate 10 mL cultures, and incubated until they reached the stationary phase. The ratio of the strains before and after competition was determined by plating 10^6 dilutions of the initial mixture and of each of the end cultures on LB agar, and the number of red- and green- fluorescent CFUs was counted. The relative fitness (W) of the mKate strain was then calculated by the following formula (14), where R_0 is the frequency of mKate before the competition, R_1 is the frequency of mKate after the competition, and F is the fold increase of bacteria during the competition as determined by the dilution:

$$W = \frac{\ln\left(\frac{R_1 \times F}{R_0}\right)}{\ln\left(\frac{(1-R_1) \times F}{1-R_0}\right)}.$$

We obtained $W = 0.99 \pm 0.01$, i.e., a very small or zero fitness disadvantage of the mKate-labeled strain versus the GFP-labeled strain. For the purpose of our biofilm experiment, in which selection is very strongly suppressed as explained in the main text, we can consider both variants to have the same fitness.
We also compared the fitness of mKate:RIF^R versus GFP:RIF^S strains in the same way as described above. We obtained $W_{R/S} = 0.83 \pm 0.01$, or equivalently $W_{S/R} = 1.20 \pm 0.01$, in good agreement with direct growth rate measurements from the previous section.

Comparison between the two- and one-step RIF protocols (SI Fig. S10).

In addition to the experiment from Fig. 4, in which we increased the concentration of RIF in two steps, we performed an experiment (three biological replicates) with a single step of RIF at 4-4.5 ug/ml (concentration accurate to about 10% as determined via light absorbance at the absorption peak of RIF, $\lambda = 475$ nm). This concentration was very close to the MIC of the sensitive strain and therefore small variations in the concentration could have had large effects on its growth rate.
Biofilms grew with slightly different rates in different wells (SI Fig. S6), possibly because of differences in the depth of the wells of low- and high- amplitude wells, or an asymmetric flow rate in the main channel. We hypothesize that this caused rifampicin to affect the biofilms

differently, potentially affecting the strength of selection differently in high vs low-amplitude wells. This made it difficult to directly compare wells with the same period T and different amplitudes. Since we chose the concentration of RIF to induce strong selective pressure in high-amplitude wells, we focused only on those wells in the subsequent analysis.

SI Figure S10A-B shows the sensitive clonal fraction, separately for each replicate. The difference between the replicates is caused by different initial fractions of resistant to sensitive cells in the wells prior to the exposure to RIF, determined by counting cells as in "*The initial distribution of bacteria*": resistant:sensitive = 0.36 ± 0.01 (replicate 1), 0.14 ± 0.01 (replicate 2), and 0.28 ± 0.01 (replicate 3). Nevertheless, all replicates show the same trend as visible in Fig. 4: selection is attenuated as T increases. The effect is even stronger than in Fig. 4, except for $T = 10$ μm and $A = 1.7$ μm.

We think the reason for this disagreement is that the single-step protocol leads to a more rapid expansion of resistant sectors than in the two-step protocol. This can be seen by comparing the plots of the sensitive clonal fraction from Fig. 4C and SI Fig. S10A-B: the sensitive clonal fraction decreases more rapidly in the 1-step than in the 2-step experiment. This probably causes the sensitive strain to be eliminated from $T = 10$ μm wells which are quite shallow ($A = 1.7$ μm) compared to other wells.

Supplementary Figures

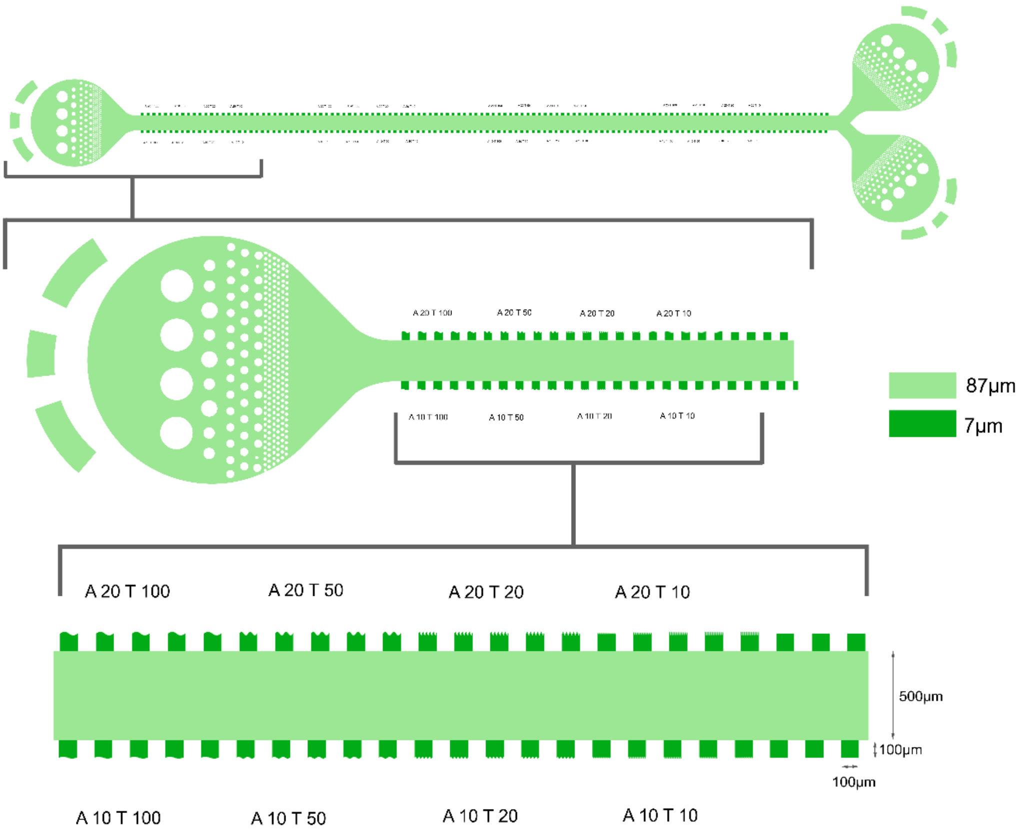

Fig. S1. Design of the microfluidics device. Top panel: the entire device. Middle and bottom panels: small sections of the main channel (width = 500 μm) with 100x100 μm wells of different types (corrugation period and amplitude) visible on both sides. T, A are in μm.

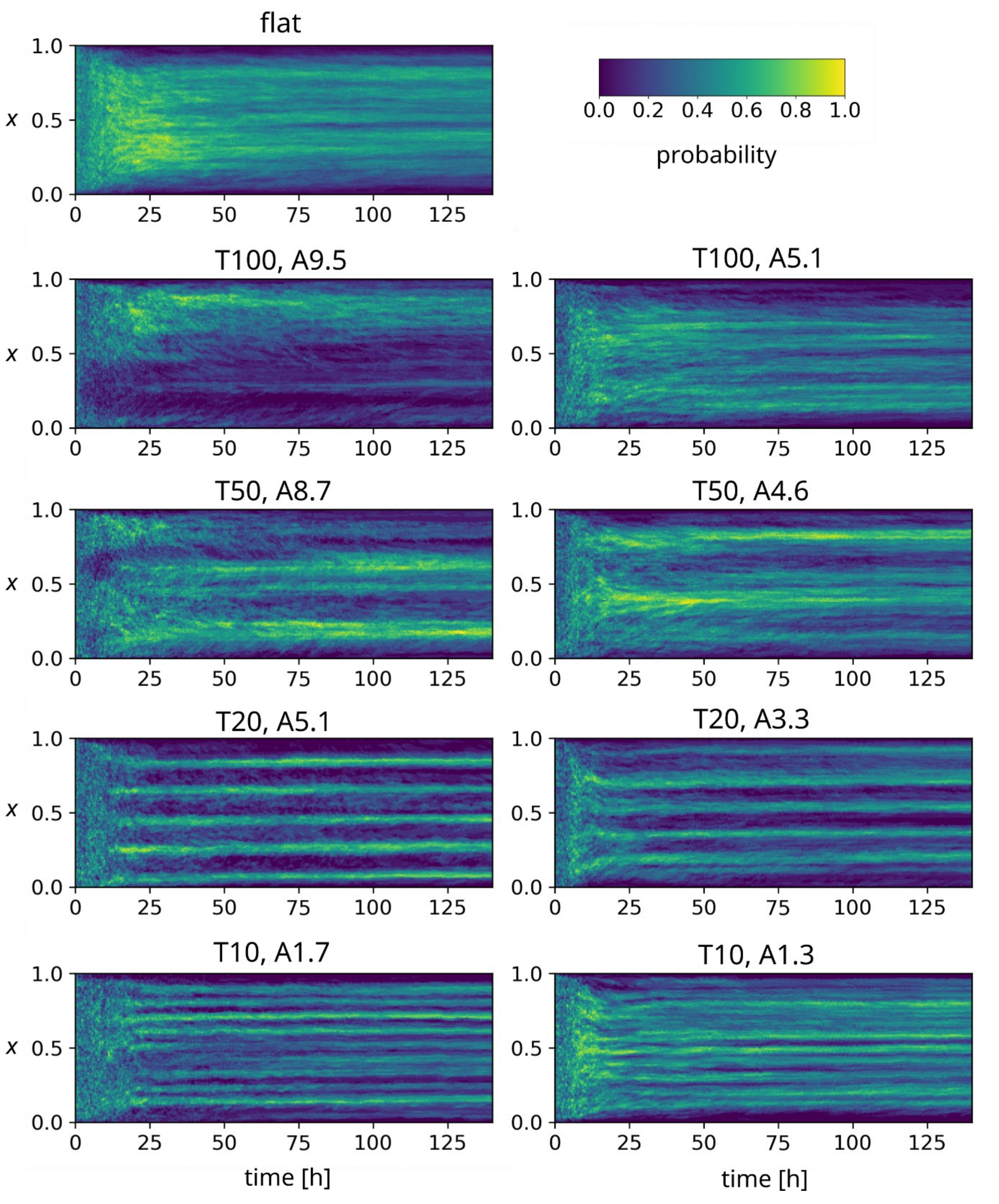

Fig. S2. Probability density of finding a sector boundary at position x. The distributions, averaged over different wells of the same type, have been plotted as a function of time. Only replicate experiment 1 is shown here. Position x within the well has been divided by the width of the well to be always between 0 (left wall) and 1 (right wall). T, A are in µm.

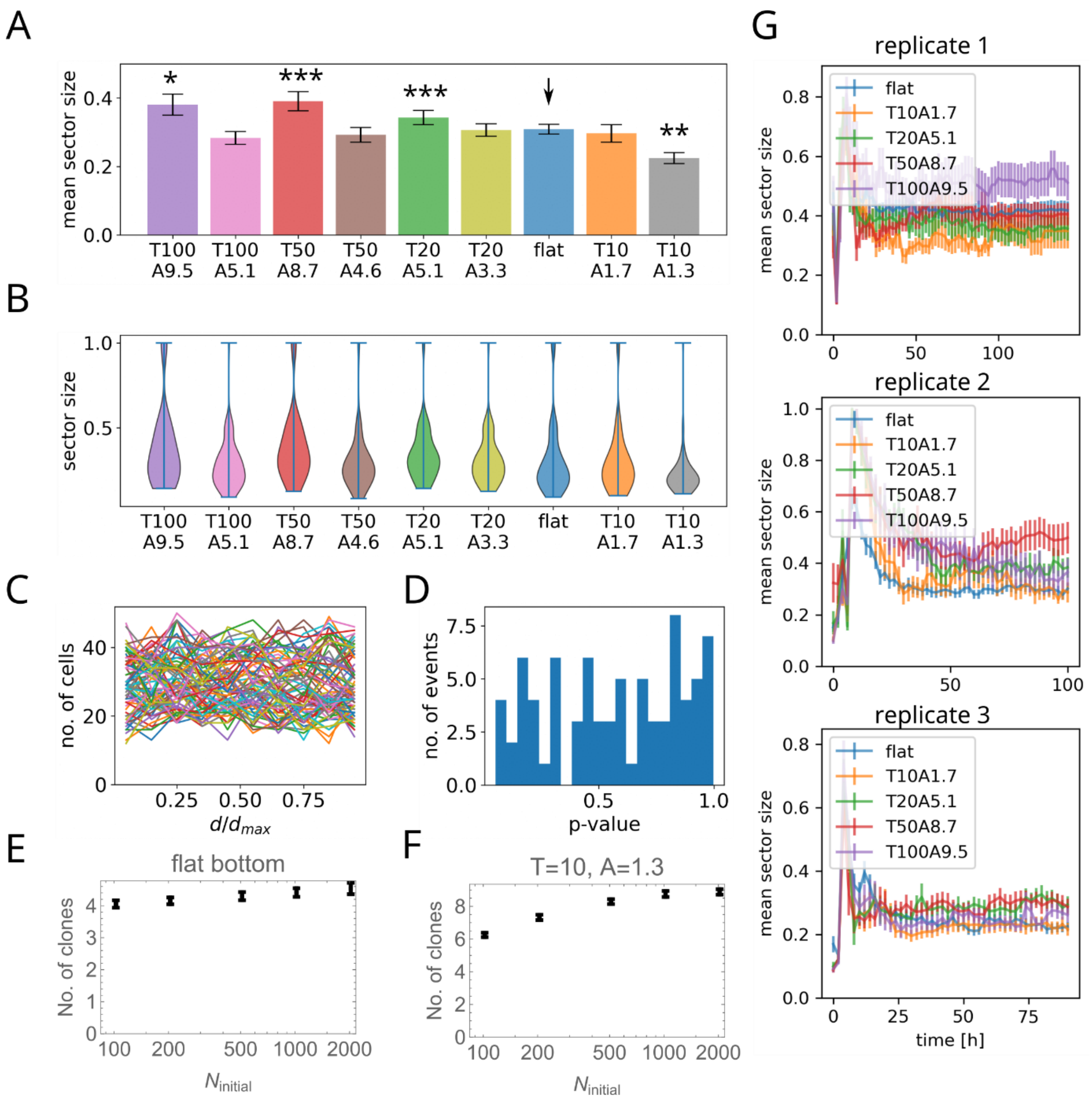

Fig. S3. Statistical analysis of data from Fig 1. (**A, B**) Mean sector size (A) and probability distributions of sector sizes (B) for different well types at the end of the experiment. Stars above the bars denote different significance levels (* = $p < 0.05$, ** = $p < 0.01$, *** = $p < 0.001$) from the Kolmogorov-Smirnov (KS) test comparing the distributions to the distribution for flat-bottom wells (indicated by a black arrow). P-values from these tests are 0.01, 0.98, 0.0001, 0.23, 0.0005, 0.06, 1.0 (flat), 0.92, 0.002, respectively. **(C)** Distribution of the number of cells at $t = 0$ (inoculation) in the wells as a function of distance d from the bottom, normalized (divided) by the maximum distance d_{max}. Bin size = 0.1. **(D)** Distribution of p-values from the KS test against a null hypothesis that the distributions from (C) are uniform. **(E, F)** Computer simulation: mean number of clones after $t = 72$ h in flat- and $T = 10, A = 1.3$ corrugated wells, for different initial number of cells. **(G)** Mean sector size for different wells as a function of time, for three biological replicates. Error bars are S.E.M., T, A are in μm.

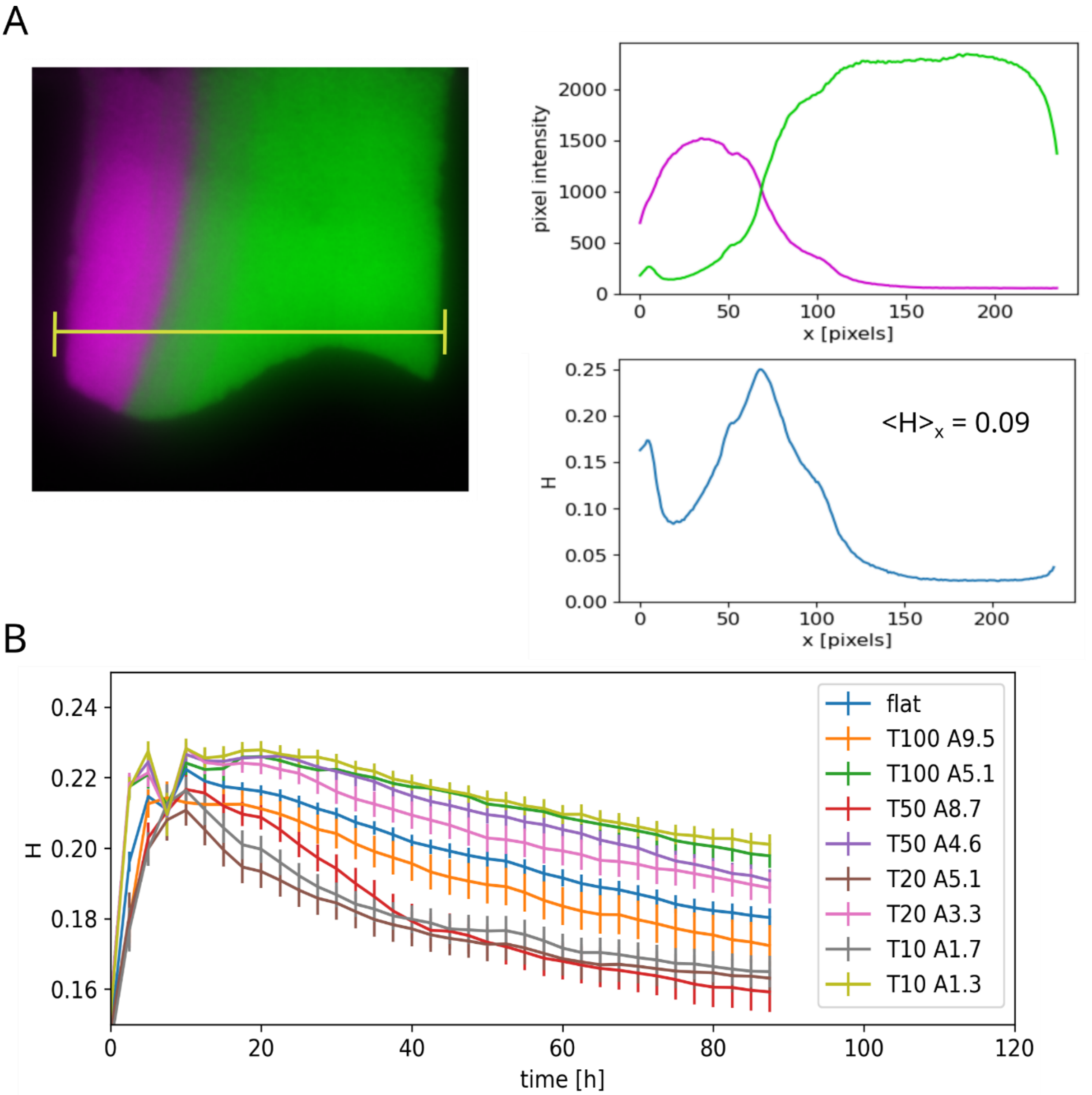

Fig. S4. Heterozygosity in wells of different types. (A) Illustration of the method with which heterozygosity is quantified. Fluorescence intensities $f_1(x), f_2(x)$ obtained from raw images by selecting lines of pixels parallel to the bottom of the wells are first normalized to account for different average fluorescence intensities of the mKate and GFP strains. We then calculate heterozygosity $H(x) = f_1(x)f_2(x)/(f_1(x) + f_2(x))$ for each position (each pixel) along the line, and finally average it for all positions x. **(B)** Heterozygosity H versus time for different types of wells, averaged over all wells of the same type, and three replicate datasets from Fig. 1. Error bars are S.E.M., T, A are in μm.

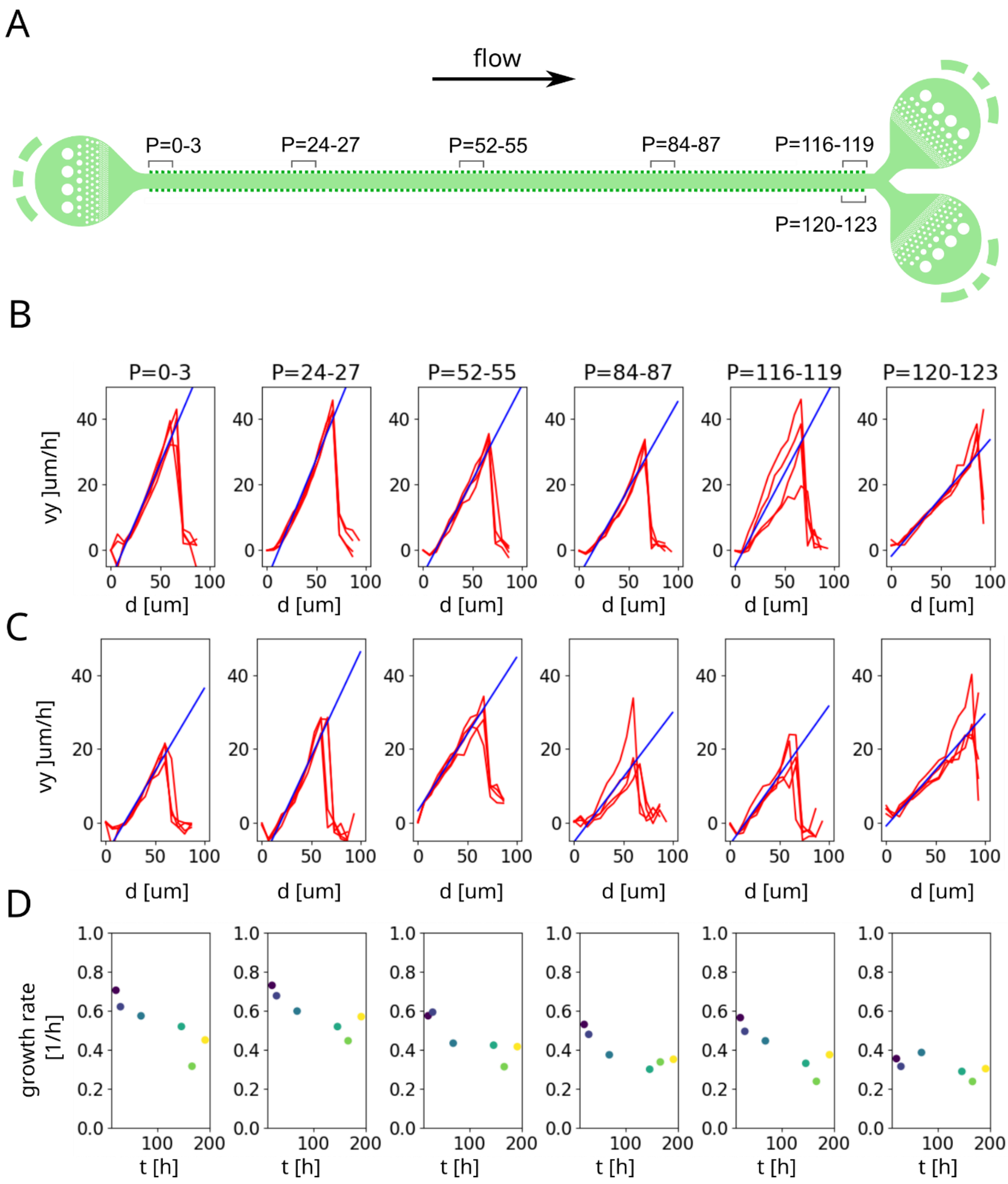

Fig. S5. Growth rates in different wells and different times, for the experiment from Fig. 1. (A) Positions of analyzed wells in the microfluidic chip (inlet on the left, outlets on the right). **(B, C)** Vertical component $v_y(d)$ of the velocity field versus distance d from the bottom, for early ($t = 20$ h) and old ($t = 192$ h) biofilm. Each panel shows four wells (red curves); the blue line is a linear fit to all four curves. **(D)** Growth rate in the same wells as in (B, C), as a function of time, obtained from linear fits to $v_y(d)$.

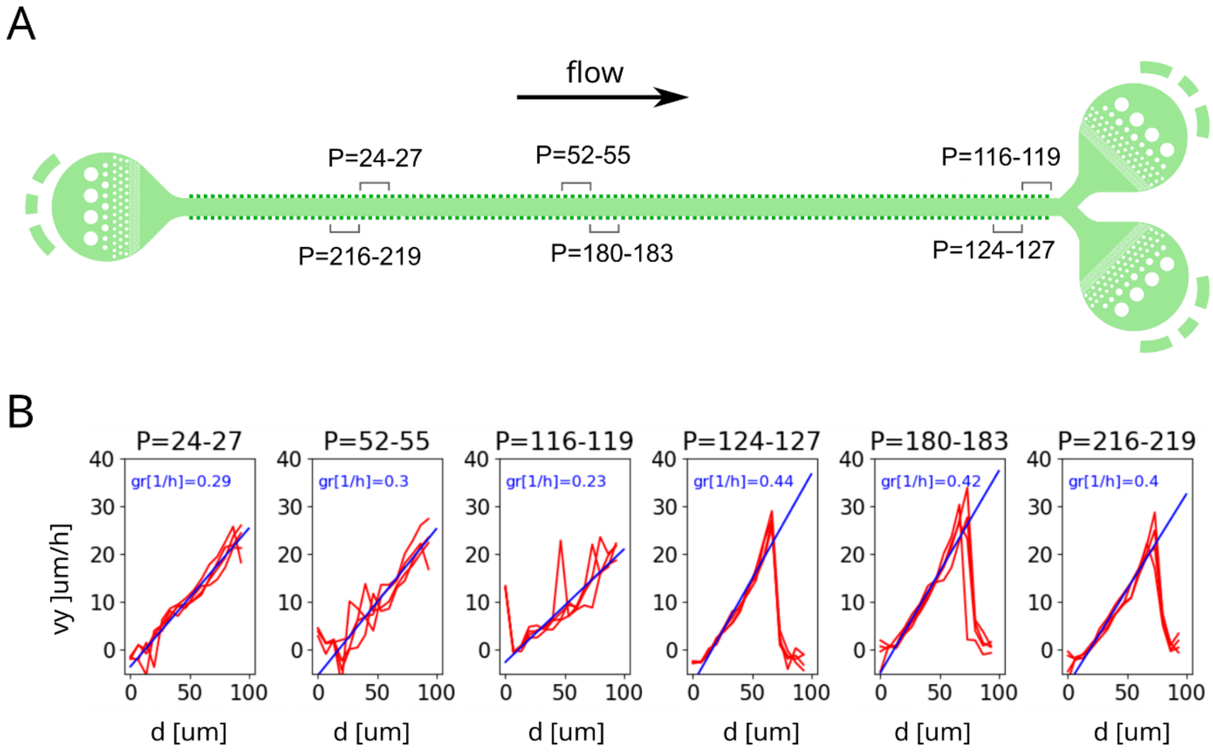

Fig. S6. Vertical component of the velocity field, for biofilms from Fig. S10, just before RIF. (A) Positions of the analyzed wells in the microfluidic chip. **(B)** Velocity $v_y(d)$ as a function distance d from the bottom. Each panel shows four wells (red curves); the blue line is a linear fit to all four curves. Growth rate (slope of the linear fit, units: h^{-1}) is shown is blue above the plots.

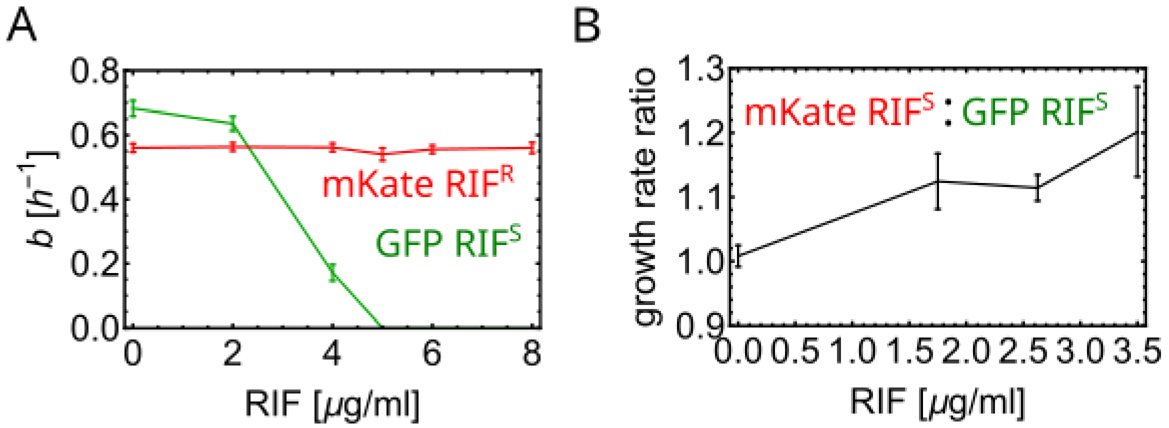

Fig. S7. **(A)** Growth rates of the RIF-sensitive GFP strain, and the RIF-resistant mKate strain. In the absence of RIF, the GFP strain grows ≈20% faster compared to the mKate strain. **(B)** The calculated ratio of the growth rates of mKate and GFP sensitive strains is consistent with no fitness difference in the absence of RIF, and a small growth advantage of mKate strain for non-zero RIF concentrations. Error bars are S.E.M.

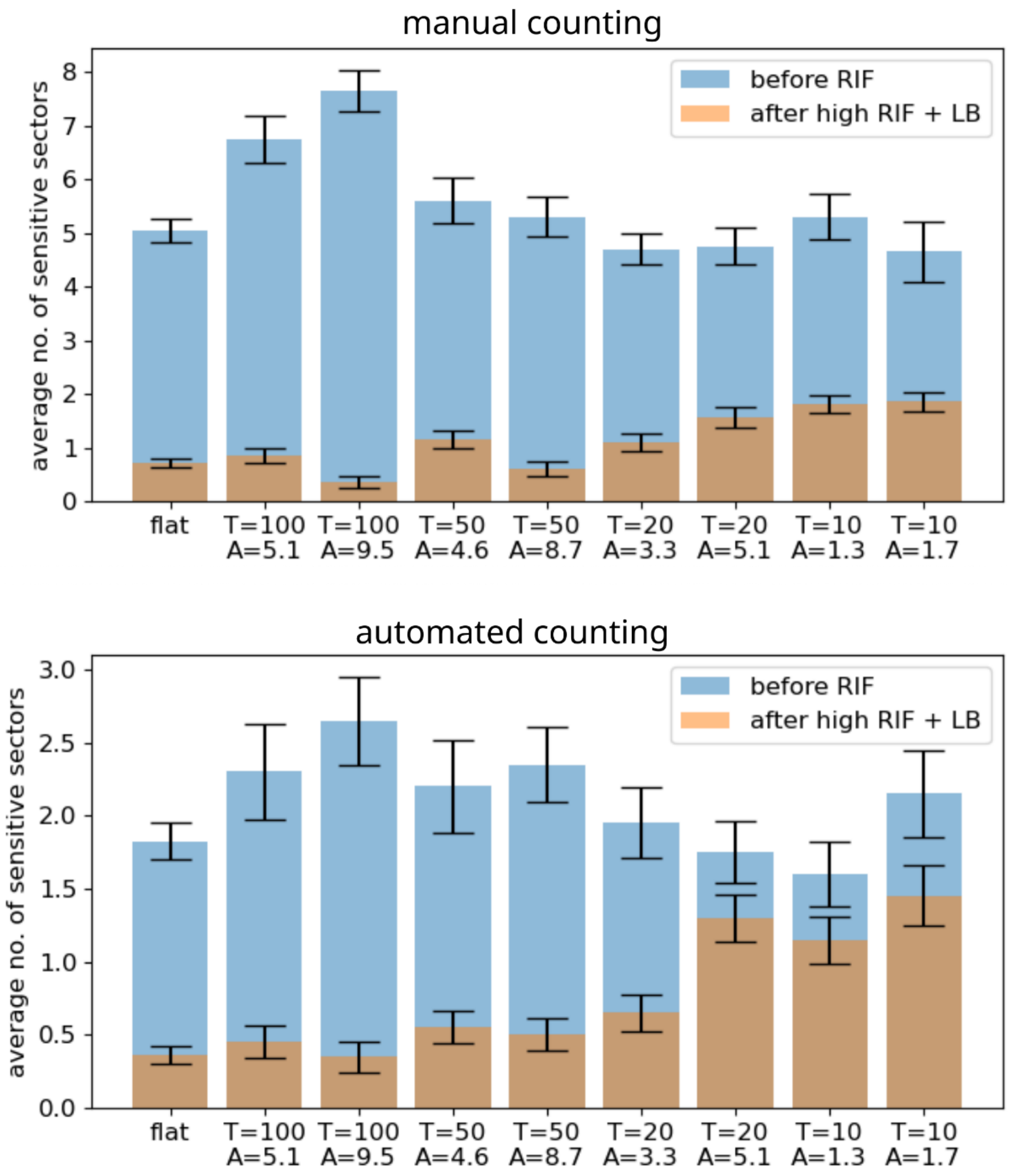

Fig. S8. Average number of green sectors in the experiment from Fig. 4. Upper panel: sectors counted manually, lower panel: sectors counted by a computer algorithm. "before RIF" = before low-RIF treatment ($t = 41$ h), "after high RIF + LB" = at the end of the experiment ($t = 182$ h). The automated algorithm deliberately ignores very small sectors, thus the numbers are generally lower than with manual counting. However, both methods show the same trend: the ratio of the number of sectors after and before the RIF exposure increases with decreasing T. Error bars are S.E.M., T, A are in μm.

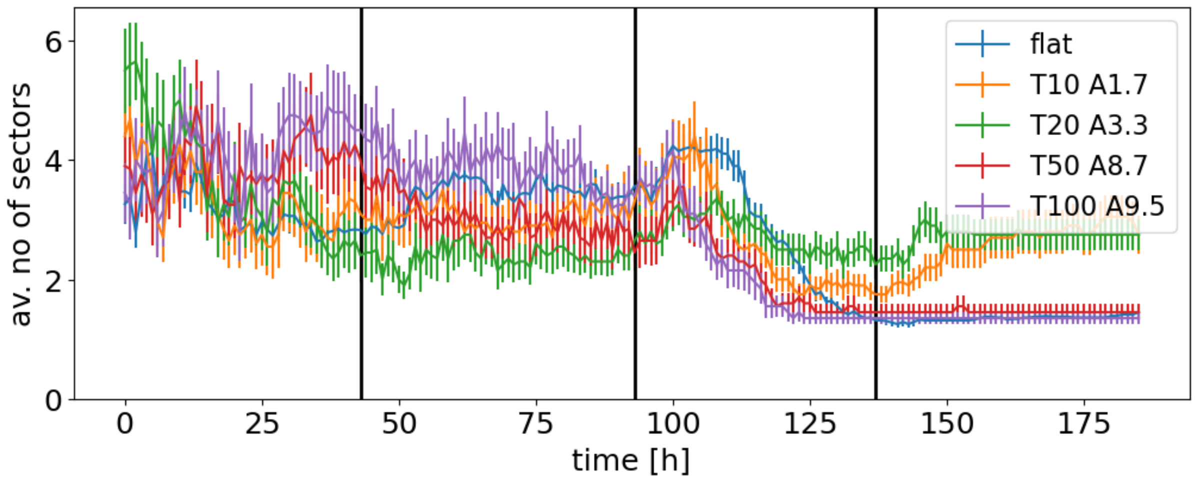

Fig. S9. Average number of sectors versus time, for the experiment from Fig. 4. The sectors have been counted using the same automated algorithm as in Fig. S8. Error bars are S.E.M., T, A are in μm.

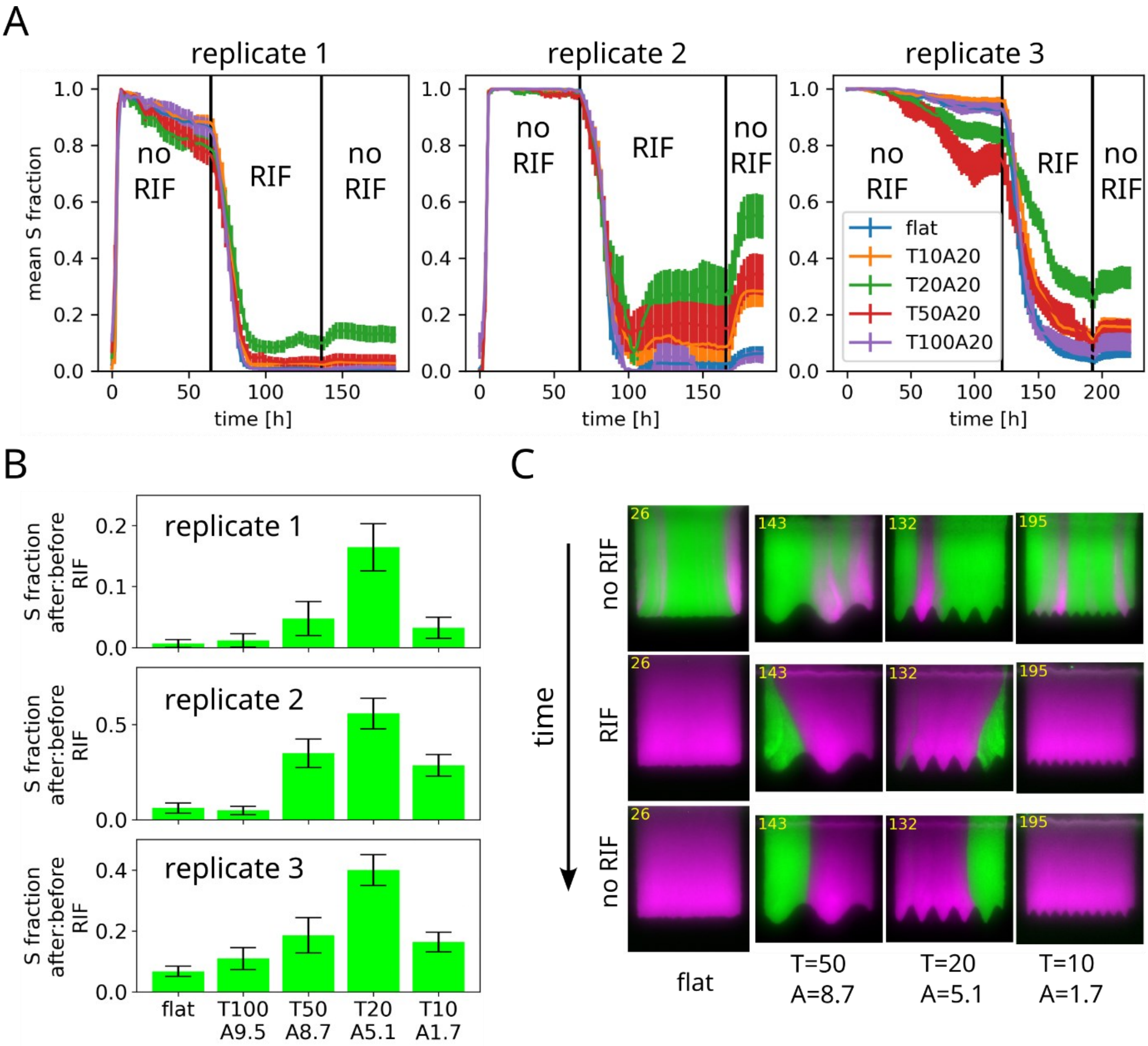

Fig. S10. A single-concentration rifampicin exposure protocol yields similar results to the two-step protocol from Fig. 4. (A) Mean sensitive fraction as a function of time, for different well types and three replicate experiments. In all cases, LB was replaced by RIF at 4-4.5 μg/ml after fluorescent sectors stabilized. RIF was replaced again by LB when the sensitive fraction stabilized. **(B)** The sensitive fraction at the end of the experiment, divided by the fraction at the start of RIF treatment. Note the difference in the vertical scale in all replicates. **(C)** Representative snapshots of different wells at different times (end of LB, end of RIF, end of experiment), for replicate 1. Magenta = resistant strain, green = sensitive strain. All error bars are S.E.M., T, A are in μm.

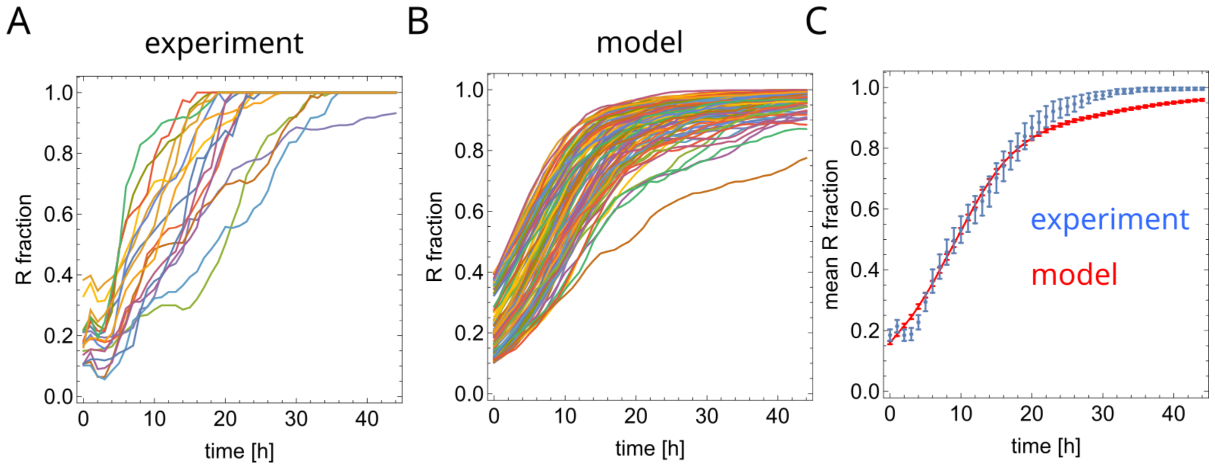

Fig. S11. Relative fitness from sector expansion. (A) The fraction of fitter (resistant, "R") cells versus time in flat-bottomed wells (colors = different wells) during the 3.5 μg/ml RIF exposure experiment from Fig. 4C. Only wells in which the initial fraction at $t = 0$ h is between 0.1 and 0.4 have been selected. **(B)** The same fraction of fitter cells from the computer model, for the relative fitness of green (sensitive) to red (resistant) cells $W_{S/R} = 0.2$, initial fraction of fitter cells having the same distribution as in panel (A) and the doubling time of 3 h. **(C)** Mean fraction of resistant cells for the experiment and the model, for $W_{S/R} = 0.2$, which gives the best fit to the experimental data. Error bars are S.E.M.

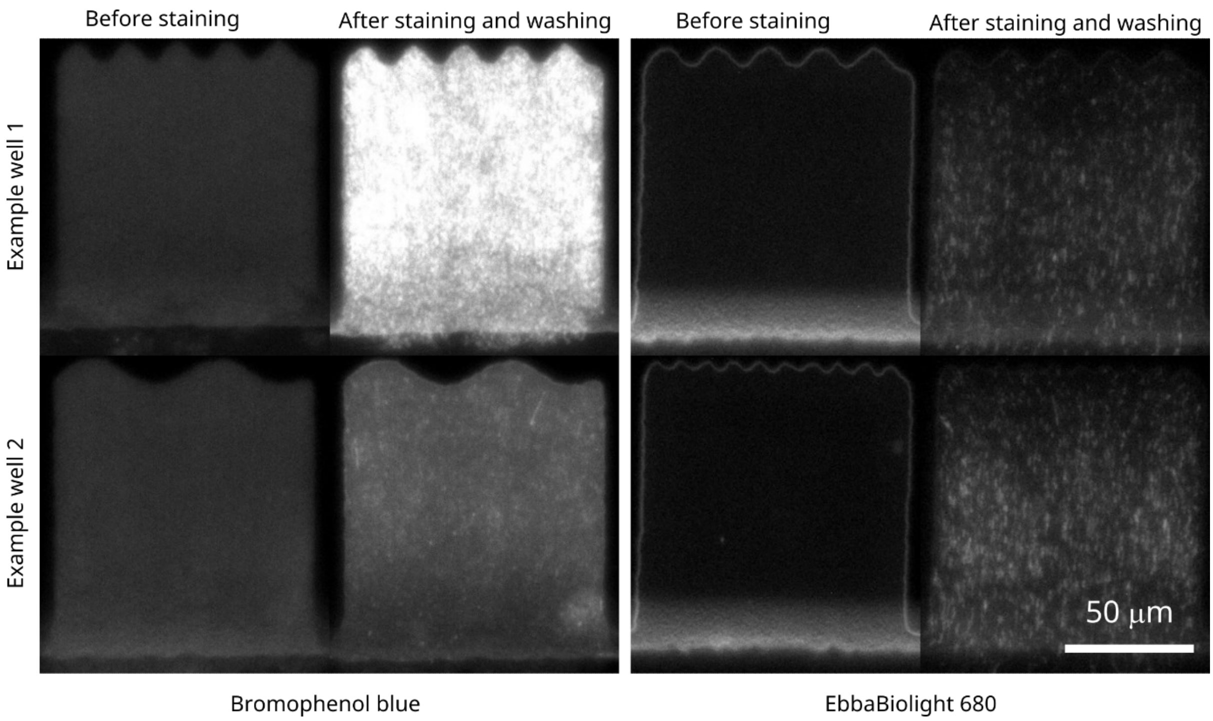

Fig. S12. The biofilm stain with bromophenol blue dye and EbbaBiotech 680. Examples of wells before and after staining. We chose wells that contained only green-fluorescent cells so as not to interfere with the red fluorescence of the dyes.

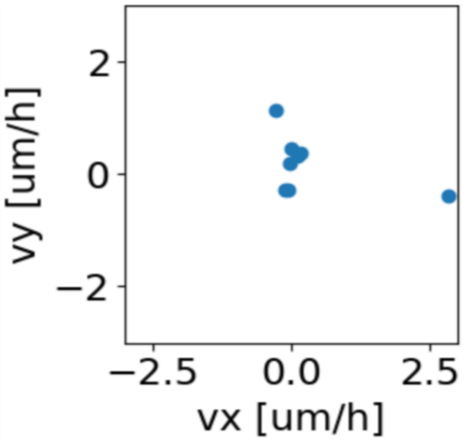

Fig. S13. Movement of a microfluidic device during imaging. The plot shows horizontal and vertical components v_x, v_y of the registered movement of the PDMS parts of the device near the biofilms from Fig. 2. Each point corresponds to a single field of view (FOV).

Supporting Videos

Movie S1 (separate file). Biofilm growth in selected wells (as in Fig. 1) from inoculation (t=0) until t=144 h.

Movie S2 (separate file). Biofilm growth in 4 wells of different type (as in Fig. 4): flat, (T,A)=(10,1.7), (T,A)=(20,5.1), and (T,A)=(50,8.7) μm, from inoculation (t=0) until t=182 h.

Movie S3 (separate file). Simulated biofilm in a flat-bottom 80x70 μm well, for the same parameters as simulations presented in Fig. 4A.

Movie S4 (separate file). Simulated biofilm in a flat-bottom 80x30 μm well, for the same parameters as Movie S3.

Movie S5 (separate file). Simulated biofilm in a flat-bottom 80x30 μm well, for the same parameters as Movie S3, with the exception of the friction coefficient being 20x larger.

SI References

1. W. Postek, K. Staskiewicz, E. Lilja, B. Waclaw (2023) https://github.com/Dioscuri-Centre/biofilms_on_corrugated_surfaces. (GitHub).
2. D. Qin, Y. Xia, G. M. Whitesides, Soft lithography for micro- and nanoscale patterning. *Nature Protocols* **5**, 491-502 (2010).
3. A. J. Link, D. Phillips, G. M. Church, Methods for generating precise deletions and insertions in the genome of wild-type Escherichia coli: application to open reading frame characterization. *Journal of Bacteriology* **179**, 6228-6237 (1997).
4. C. Merlin, S. McAteer, M. Masters, Tools for Characterization of *Escherichia coli* Genes of Unknown Function. *Journal of Bacteriology* **184**, 4573-4581 (2002).
5. S. Jaramillo-Riveri *et al.*, Growth-dependent heterogeneity in the DNA damage response in Escherichia coli. *Mol. Syst. Biol.* **18**, e10441 (2022).
6. D. D. Yang *et al.*, Fitness and Productivity Increase with Ecotypic Diversity among Escherichia coli Strains That Coevolved in a Simple, Constant Environment. *Appl. Environ. Microbiol.* **86:**e00051-20 (2020).
7. A. Edelstein, N. Amodaj, K. Hoover, R. Vale, N. Stuurman, Computer Control of Microscopes Using µManager. *Current Protocols in Molecular Biology* **92**, 14.20.11-14.20.17 (2010).
8. B. K. P. Horn, B. G. Schunck, Determining optical flow. *Artificial Intelligence* **17**, 185-203 (1981).
9. A. Dal Co, S. van Vliet, M. Ackermann, Emergent microscale gradients give rise to metabolic cross-feeding and antibiotic tolerance in clonal bacterial populations. *Phil. Trans. R Soc. London B Biol. Sci.* **374**, 20190080 (2019).
10. D. Volfson, S. Cookson, J. Hasty, L. S. Tsimring, Biomechanical ordering of dense cell populations. *Proceedings of the National Academy of Sciences* **105**, 15346-15351 (2008).
11. H. H. Tuson *et al.*, Measuring the stiffness of bacterial cells from growth rates in hydrogels of tunable elasticity. *Mol. Microbiology* **84**, 874-891 (2012).
12. L. Wang, D. Fan, W. Chen, E. M. Terentjev, Bacterial growth, detachment and cell size control on polyethylene terephthalate surfaces. *Sci. Rep.* **5**, 15159 (2015).
13. N. Ojkic *et al.*, A Roadblock-and-Kill Mechanism of Action Model for the DNA-Targeting Antibiotic Ciprofloxacin. *Antimicrob. Agents Chemother.* **64**:10.1128/aac.02487-19 (2020).
14. M.-C. Lee, H.-H. Chou, C. J. Marx, Asymmetric, bimodal trade-offs during adaptation of Methylobacterium to distinct growth substrates. *Evolution* **63**, 2816-2830 (2009).
15. L. Zhang, Z. Li, Z. Chen, Live cell fluorescent stain of bacterial curli and biofilm through supramolecular recognition between bromophenol blue and CsgA. *Chem. Commun.* **56**, 5014-5017, (2020).
